# Modular Semisynthetic Approach to Generate T Cell-Dependent Bispecific Constructs from Recombinant IgG1 Antibodies

**DOI:** 10.1021/acs.bioconjchem.4c00309

**Published:** 2024-09-16

**Authors:** Irene Shajan, Léa N. C. Rochet, Shannon R. Tracey, Rania Benazza, Bianka Jackowska, Oscar Hernandez-Alba, Sarah Cianférani, Christopher J. Scott, Floris L. van Delft, Vijay Chudasama, Bauke Albada

**Affiliations:** † Laboratory of Organic Chemistry, Wageningen University & Research, Stippeneng 4, Wageningen 6807 WE, The Netherlands; ‡ Department of Chemistry, University College London, 20 Gordon St, London WC1H 0AJ, United Kingdom; § Patrick G. Johnston Centre for Cancer Research, School of Medicine, Dentistry and Biomedical Sciences, Queen’s University Belfast, 97 Lisburn Road, Belfast BT9 7BL, United Kingdom; ∥ Laboratoire de Spectrométrie de Masse BioOrganique, Université de Strasbourg, CNRS, IPHC UMR 7178, 67000 F-Strasbourg, France; ⊥ Infrastructure Nationale de Protéomique ProFI—FR2048, 67087 Strasbourg, France; # Synaffix BV—A Lonza Company, Kloosterstraat 9, Oss 5349 AB, The Netherlands

## Abstract

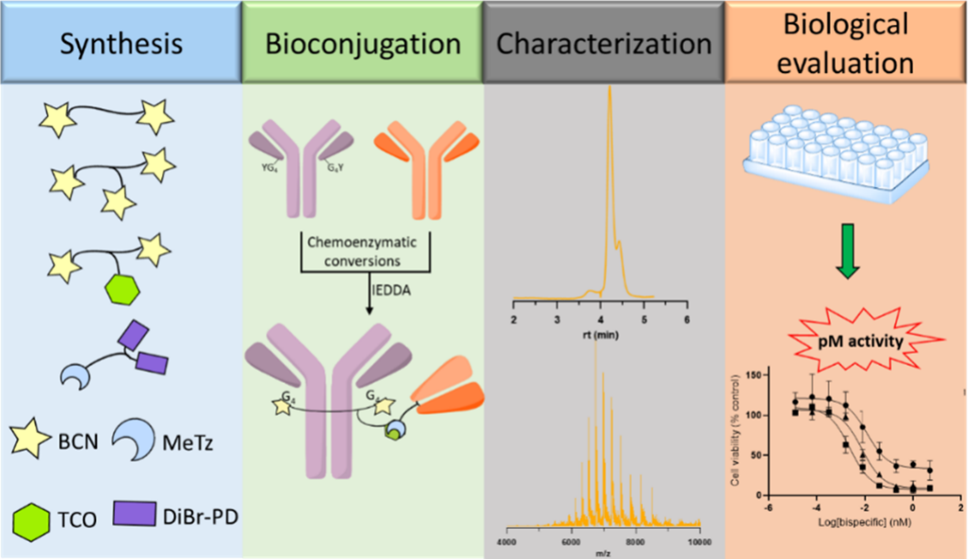

Redirecting T cells to tumor cells by bispecific antibodies is an effective approach to treat cancer, and T cell-dependent bispecific antibodies (TDBAs) are an emerging class of potent immunotherapeutic agents. By simultaneously targeting antigens on tumor cells and T cells, T cells are activated to kill tumor cells. Herein, we report a platform to generate a novel class of 2:1 structure of T cell-dependent bispecific antibody with bivalency for HER2 receptors on tumor cells and monovalency for CD3 receptors on T cells. For this, we use a biogenic inverse electron-demand Diels–Alder (IEDDA) click reaction on genetically encoded tyrosine residues to install one TCO handle on therapeutically approved antibody trastuzumab. Subsequent TCO-tetrazine click with a tetrazine-functionalized CD3-binding Fab yields a 2:1 HER2 × CD3 TDBA that exhibits a tumor-killing capability at picomolar concentrations. Monovalency toward the CD3 receptor on T cells can lower the chances of cytokine release syndrome, which is a common side effect of such agents. Our semisynthetic approach can generate highly potent TDBA constructs in a few chemoenzymatic and synthetic steps.

Monoclonal antibodies (mAbs) enjoy numerous therapeutic applications due to their ability to engage specific antigens on cell surfaces.^1−4^ Since native (homodimeric) mAbs as such have limited applications in therapy due to the absence of a curing agent,^5^ different formats of antibodies have been developed for various therapeutic applications.^6−8^ As of 2023, 145 mAb-based drugs have been approved by the United States Food and Drug Administration (US FDA).^9^ Of these, 11 bispecific antibodies have been marketed in the US.^10^ Among them, T cell-engaging constructs form the largest class in the clinical pipeline^11^ with eight bispecific T cell engagers in the market for cancer treatment.^12^ T cell engagers are antibody-based therapeutics that simultaneously engage the CD3 receptor on the T cell and a tumor-associated antigen.^13,14^ Different formats of T cell engagers have been developed, like diabody,^15^ CrossMab,^16^ BiTE,^17,18^ dual affinity retargeting antibodies (DART),^19,20^ tandem diabody (TandAb),^21^ synthetic bispecific mAbs (SynAbs),^22^ checkpoint inhibitory T cell engagers (CiTEs),^23,24^ and, more recently, bispecific antibody constructs (bsAcs).^25^ Similar to the first FDA-approved bispecific construct blinatumomab,^26^ most of these formats are based on or derived from small fragment antigen-binding regions (Fabs, typically 50–60 kDa).^27,28^ Due to their size, fragment-based bispecifics suffer from short half-life and require continuous infusion. This is less the case for IgG-like bispecifics, such as mosunetuzumab,^29,30^ which offer higher stability and longer serum half-life.^31^

Besides classical biosynthesis of bispecifics via recombinant expression, the evolution of bio-orthogonal (click) chemistry has led to the development of antibody conjugates produced by synthetic methods, also using native mAbs.^32−34^ In this approach, strain-promoted azide-alkyne cycloaddition (SPAAC),^35,36^ inverse electron-demand Diels–Alder (IEDDA) reaction between a strained alkene/alkyne and tetrazine,^37^ and alkyne strain-promoted oxidation-controlled *ortho*-quinone (SPOCQ)^38−40^ cycloaddition have been successfully employed to develop antibody conjugates. The modularity of this approach offers a late-stage combination of Fabs, which may be appealing for screening Fab-mAb combinations prior to upscaling and production.

Due to the homodimeric nature of native antibodies, a 2:1 format of a T cell-dependent bispecific is less conveniently accessible than a 2:2 conjugate, although methods for heterodimerization of antibodies have also proven useful for the preparation of 2:1 bispecifics.^41−43^ Efforts from our own group in the past have shown that a G_4_Y- or sortase-tagged knob-in-hole (KiH) antibody can be converted to a 2:1 bispecific antibody conjugate via sortase-mediated ligation, followed by an IEDDA reaction with MeTz-UCHT1.^42^ On the other hand, the first IgG-based carcinoembryonic antigen T cell bispecific antibody (CEA TCB) to enter clinical trials was generated fully recombinantly.^44^ The limited number of reports on the generation of such asymmetric constructs indicates the complexity of the process. For example, a 2:1 format of bispecific antibody was prepared from native mAbs via Ugi reaction on the N-terminus of the mAb, followed by a SPAAC reaction with a rebridged Fab fragment.^45^

Herein, we report a convenient approach in which our biogenic tyrosine-based click reaction between an oxidized tyrosine and a BCN handle is used to prepare 2:1 HER2 × CD3 T cell-dependent bispecific antibody construct from a homodimer, namely, tras[LC]G_4_Y. With a construct that displays bivalency for the tumor-associated antigen (TAA) and monovalent binding to T cells, preferential T cell activation at tumor cells over healthy cells can be retained.^46^ Specifically, it can be expected that monovalent binding to the T cell-associated CD3 receptor aids in avoiding CD3 activation in the absence of TAA.^47^ As such, we present an approach for the convenient conversion of antibodies with a C-terminally placed G_4_Y tag on light chains into an IgG-like 2:1 TDBA via a few chemical and chemoenzymatic steps.

## Results and Discussion

### Synthesis of T Cell-Dependent Bispecific Antibodies

**Scheme 1:**
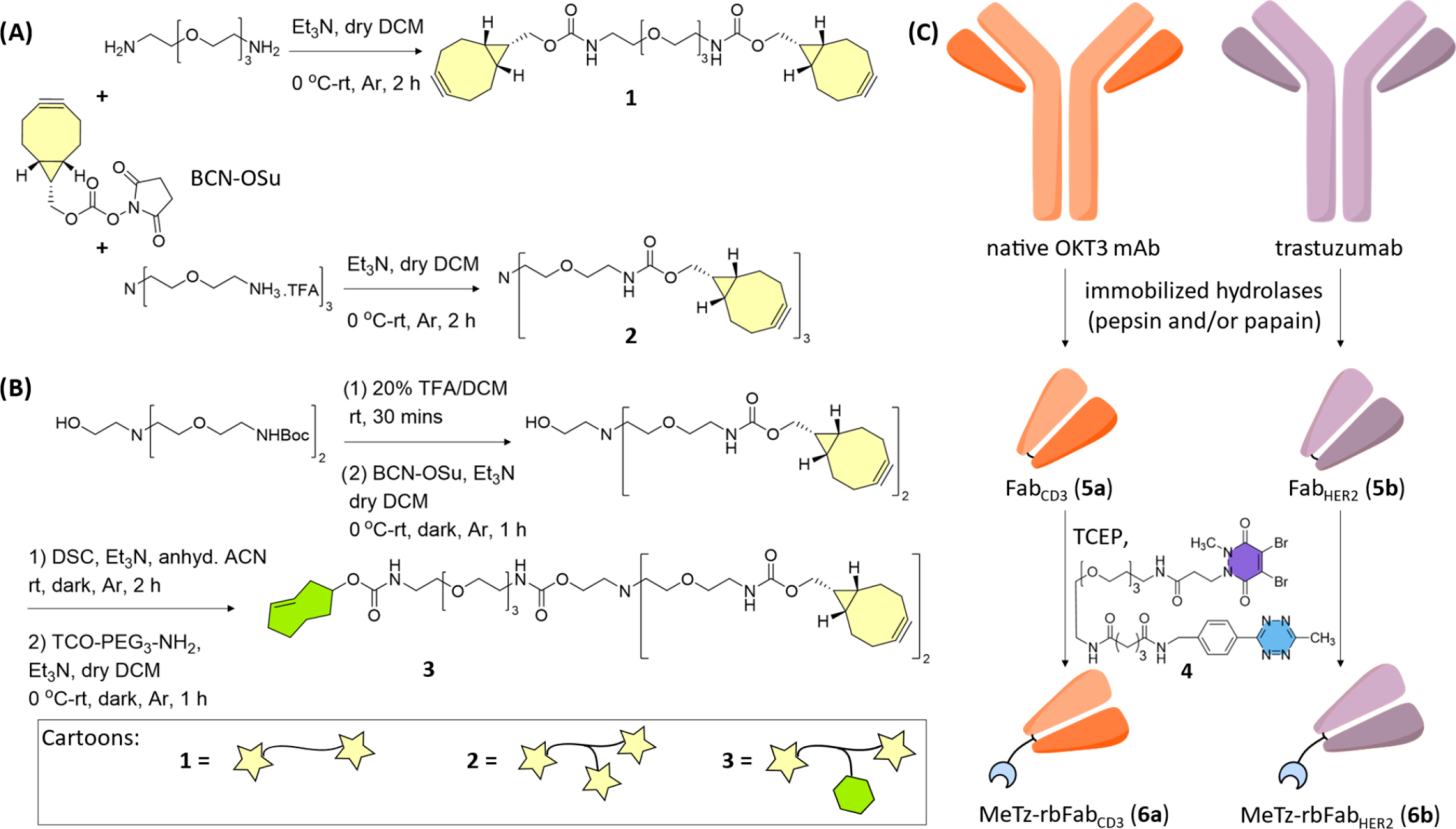
Conversion of Two mAbs into a 2:1 Bispecific Construct (A) Synthesis of bis-BCN (**1**) and tri-BCN (**2**) linkers. (B) Synthesis of the BCN_2_-TCO (**3**) linker. (C) Antibody digestion followed by Fab reduction and rebridging with TCEP in the presence of MeTz-PEG_3_-Br_2_PD (**4**), resulting in rebridged Fabs MeTz-rbFab_CD3_ (**6a**) and MeTz-rbFab_HER2_ (**6b**). Abbreviations for used reagents: DSC: *N*,*N*-disuccinimidyl carbonate, TCEP: tris(2-carboxyethyl)phosphine, and TFA: trifluoroacetic acid.

For the preparation of 2:1 bispecific antibody constructs, we synthesized a set of three different BCN-functionalized linkers and also rebridged the Fabs of two antibodies (Scheme 1). Bis-BCN linker **1** and tri-BCN linker **2** were obtained after the reaction of the corresponding PEG-diamine or PEG-triamine with BCN-OSu (Scheme 1A). Bis-BCN-mono-TCO-functionalized linker **3** was obtained after sequential incorporation of the BCN handles and TCO handle in a properly functionalized trimeric molecule (Scheme 1B). Partial antibody digestion using immobilized hydrolases and subsequent reduction of the intermolecular disulfides in the presence of MeTz-functionalized rebridging agent **4** resulted in MeTz-functionalized rebridged Fabs of the anti-CD3 antibody (**6a**) and trastuzumab (**6b**), which would function as a negative control (Scheme 1C).

**Scheme 2:**
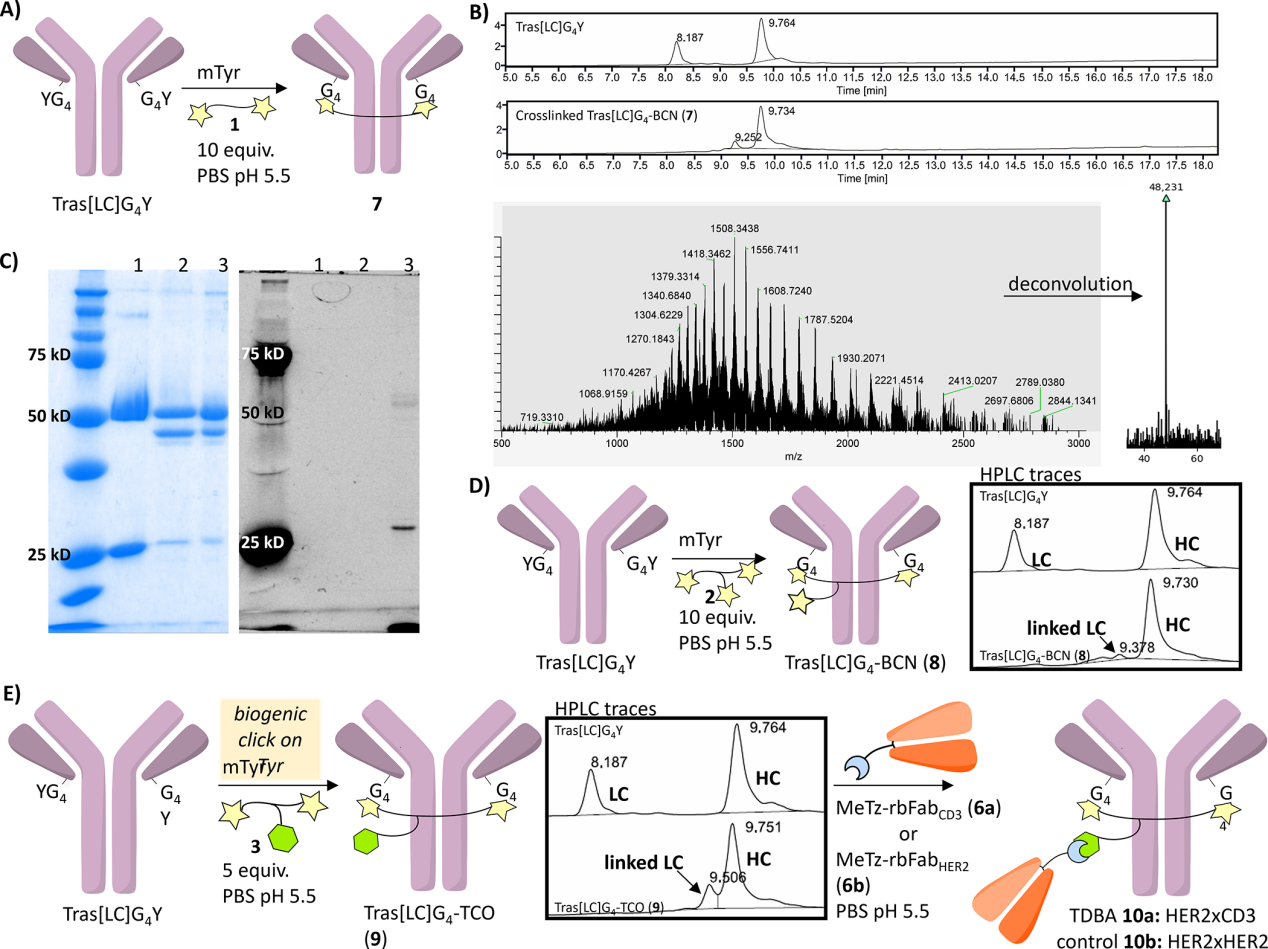
Cross-linking of the Light Chains and Assembly of the Various Modules in 2:1 Bispecific Antibody Constructs (A) Chemoenzymatic cross-linking of tras[LC]G_4_Y with bis-BCN (**1**). (B) HPLC traces of tras[LC]G_4_Y (*Top*) and product (**7**) after SPOCQ with bis-BCN **1** (*Bottom*), MS of product at *t*_R_ 9.3 min corresponds to cross-linked light chains. (C) Reducing SDS-PAGE reveals the absence of BCN in the 50 kDa band; lane 1: tras[LC]G_4_Y, lane 2: antibody construct **7**, lane 3: IEDDA of **7** with MeTz-TAMRA. (D) Chemoenzymatic cross-linking of tras[LC]G_4_Y with tri-BCN (**2**), resulting in tras[LC]G_4_-BCN (**8**). (E) Chemoenzymatic cross-linking of tras[LC]G_4_Y with BCN_2_-TCO (**3**) results in tras[LC]G_4_-TCO (**9**), which was converted to T cell-dependent bispecific HER2 × CD3 antibody (**10a**) by reaction with MeTz-rbFab_CD3_ (**6a**). Negative control construct **10b** (HER2 × HER2) was prepared analogously using MeTz-rbFab_HER2_ (**6b**).

We found that a SPOCQ-alkyne reaction on a C-terminally placed tyrosine residue in the light chains of a transiently expressed tras[LC]G_4_Y antibody with bis-BCN (BCN-PEG_3_-BCN, **1**, see Scheme 1A) yields intramolecularly cross-linked light chains (Scheme 2A,B, Figure S1, Supporting Information). The reaction of the resulting antibody construct in which the light chains were linked with MeTz-TAMRA revealed that only residue light chains became fluorescent (see band at 25 kDa in lane 3, Scheme 2C) and that the linked light chains at 50 kDa did not react with this dye.

In view of the efficiency of this conversion, we reasoned that an analogous trifunctional linker, tri-BCN (**2**, see Scheme 1A), with identical distance between the BCN handles as for bis-BCN **1**, should be able to generate an antibody with one free BCN handle. Indeed, the reaction of tras[LC]G_4_Y with tri-BCN linker **2** resulted in interconnected light chains (Scheme 2D), which did react with MeTz-TAMRA (see Figure S2, Supporting Information). Assessment of the effect of the number of equivalents on cross-linking efficiency revealed that cross-linking was achieved over the range of 0.5–10 equiv., although mostly compromised by the formation of higher oligomers and functionalization of each light chain with one BCN handle, except when the substoichiometric amounts were applied (see Figure S2E, Supporting Information). It appeared that the tri-BCN construct **2** yielded poor control over the outcome of cross-linking and presented a suboptimal route to the formation of 2:1 bispecific constructs despite the convenience associated with the synthesis of the tri-BCN linker.

To counter this, we redesigned our linker by substituting one BCN moiety for a TCO handle (Scheme 1B). Since BCN has 150-fold higher reactivity with an *ortho*-quinone when compared to TCO,^48^ we reasoned that the presence of a TCO handle instead of one of the BCN handles would favor cross-linking of the two light chains and lead to clean production of the TCO-functionalized mAb. On top of this, as TCO reacts faster with tetrazine than BCN,^49^ we expected that construction of our targeted bispecifics would be expedited. Therefore, we synthesized a BCN_2_-TCO (**3**) linker (Scheme 1B), in which the spacer length between the two BCN handles was identical to the distance in our tri-BCN construct (**2**). Subsequently, tras[LC]G_4_Y was incubated with mTyr and BCN_2_-TCO **3** (5 equiv) at 4 °C for 16 h (Scheme 1E). Indeed, a SPOCQ-alkyne reaction of the tras[LC]G_4_Y with BCN_2_-TCO linker **3** resulted in the installation of a TCO handle on the mAb by intramolecularly cross-linked light chains of tras[LC]G_4_Y via the two BCN units in linker **3** (see Figure S3, Supporting Information).

As expected, the formation of higher oligomers was suppressed substantially, especially when at least 10 equiv. of linker **3** was used. Importantly, a SPAAC reaction between TCO-functionalized mAb and azido-TAMRA did not yield a fluorescent product, confirming our assumption that BCN outcompetes TCO in the SPOCQ reaction on the oxidized tyrosine residues and that the mAb was now functionalized with a TCO handle, which does not rapidly react with an organic azide.

For the generation of MeTz-rbFab_CD3_ (**6a**), OKT3 mAb (anti-CD3 mAb) was first treated with immobilized papain resulting in two Fab units that were rebridged with MeTz-PEG_3_-Br_2_PD (**4**) after treatment with TCEP, as was described before (Scheme 1B, see the Supporting Information for details, Figures S4–S10).^22,23,25,35^ Having prepared tetrazine-functionalized Fab fragments of both OKT3 mAb and HER2 mAb, i.e., MeTz-rbFab_CD3_**6a** and MeTz-rbFab_HER2_**6b**, we performed the IEDDA reaction on tras[LC]G_4_X-TCO (X = SPOCQ product between tyrosine and BCN). For this, the TCO-functionalized antibody was incubated with MeTz-rbFab_CD3_ (**6a**) or MeTz-rbFab_HER2_ (**6b**) (0.9 equiv) at 4 °C for 2 h in PBS, pH 5.5 (Scheme 2E, see Supporting Information Figures S11A and S12A). Analysis of the reaction mixture using non-reducing SDS-PAGE revealed the successful formation of the conjugate, after which the desired product was purified using protein A purification and subsequent size exclusion chromatography (see Supporting Information, Figures S11B,C and S12B,C). The cleanest fractions of each SEC purification were collected for characterization and biological evaluation.

**Figure 1:**
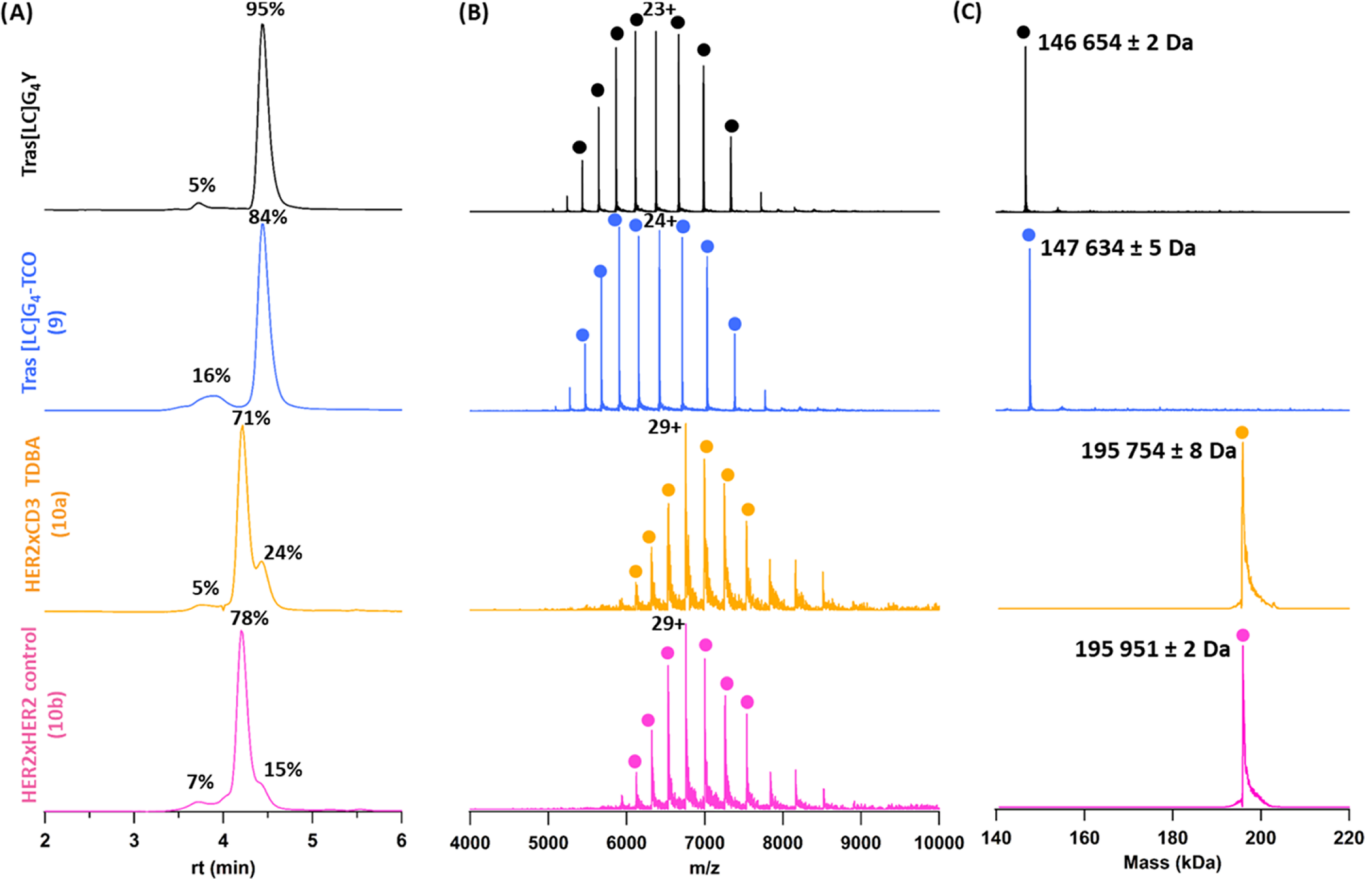
SEC-nMS analysis after endo S trimming of mAb samples obtained during bispecific formation via bio-orthogonal tyrosine-based click chemistry. (A) SEC-UV chromatogram tras[LC]G_4_Y, tras[LC]G_4_-TCO **9**, HER2 × CD3 TDBA **10a**, and HER2 × HER2 bispecific construct **10b** (from top to bottom). Relative quantification of each species is performed upon the integration of chromatographic peak areas. (B) nMS spectra of the major peak from each sample, namely, tras[LC]G_4_Y, monomer of tras[LC]G_4_-TCO **9**, 2:1 HER2 × CD3 TDBA **10a**, and 2:1 HER2 × HER2 bispecific construct **10b**. (C) Deconvolution of the main peaks, showing their corresponding masses. The experimental masses of all other species are provided with standard deviations obtained from at least four different charge states.

For characterization of the formed species and assessment of the purity of the collected fractions, size exclusion chromatography-native mass spectrometry (SEC-nMS) was performed on both the HER2 × CD3 (**10a**) and HER2 × HER2 (**10b**) conjugates (Figure 1). This revealed that even after protein A and SEC purification, we were not able to fully isolate the targeted compounds as pure fractions. Specifically, the SEC-UV chromatograms revealed the presence of a minor amount of earlier eluting species (around 3.6 min) and a more significant later eluting shoulder on the peak of the main compound at 4.4 min. MS analysis revealed that this peak corresponds to unreacted tras[LC]G_4_-TCO (**9**), which is attributed to our use of substoichiometric amounts of MeTz-rbFab (**6a**/**6b**) in the preparation of 2:1 constructs. Gaussian fitting of the partially coeluting species yielded relative intensity of 71% of 2:1 HER2 × CD3 TDBA (**10a**) in the purified fraction while 78% for 2:1 HER2 × HER2 construct (**10b**).

Therefore, the SEC-nMS reveals that obtained fractions mostly contained the targeted constructs, with relative intensities of leftover starting material tras[LC]G_4_-TCO (**9**) of 24 and 15% in **10a** and **10b**, respectively.

### Biological Evaluation

**Figure 2:**
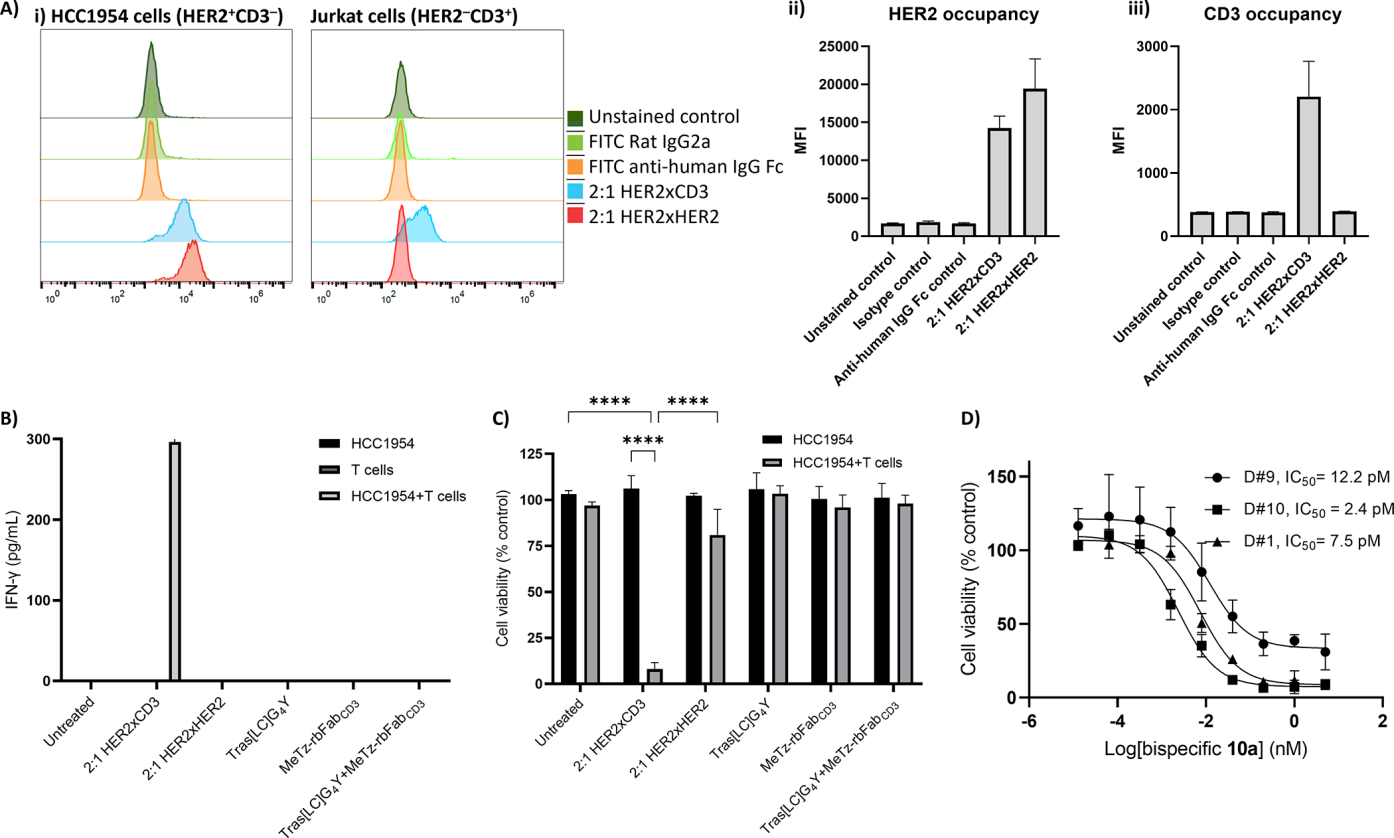
Biological activity studies of the bispecific antibody construct and controls. (A) (i) Flow cytometry analysis of binding of the various constructs to HCC1954 (HER2^+^CD3^–^) and Jurkat (HER2^–^CD3^+^) cells, (ii) binding of the constructs to HCC1954 (HER2^+^CD3^–^) cells shown as mean fluorescence intensity (MFI), and (iii) binding of the constructs to Jurkat (HER2^–^CD3^+^) cells shown as MFI (*n* = 3). (B) Induction of IFN-γ production and excretion by the various constructs and controls (5 nM) in T cells or HCC1954 (HER2^+^CD3^–^) cells alone or HCC1954/T cell cocultures (ratio 1:10). Culture supernatant IFN-γ was quantified by ELISA at 48 h following treatment. (C) Cellular metabolism assay as a measure of HCC1954 cell viability affected by the synthetic constructs in the presence of HCC1954 (HER2^+^CD3^–^) cells alone or HCC1954/T cell cocultures (E/T ratio 10:1, 5 nM construct). HCC1954 viability was assessed by Cell Titer-Glo at 48 h following treatment. (D) Cytotoxicity dose–response curve of the bsAc **10a** on HCC1954/T cell cocultures (ratio 1:10) was incubated with varying concentrations (serial dilutions ranging from 0.0128 pM to 5 nM; donors are indicated with D#1, D#9, D#10). HCC1954 cell viability was assessed by a Cell Titer-Glo at 48 h following treatment, where the IC_50_ value was extrapolated. Statistical analysis was performed in GraphPad Prism (v9.5.1), where data is presented at mean ± SEM. Statistical significance was established by Two-way ANOVA and Šídák’s multiple comparison tests (**** *p* ≤ 0.0001).

For the evaluation of the biological activity of the constructs, we first determined the binding capability of the synthesized bispecific constructs: the 2:1 HER2 × CD3 construct and the 2:1 HER2 × HER2 negative control, by means of flow cytometry. For this, we used HCC1954 (HER2^+^CD3^–^) tumor cells and Jurkat (HER2^–^CD3^+^) cells (Figure 2A). In this study, HCC1954 cells were incubated with either of the synthesized bispecific constructs and then stained with the FITC-labeled anti-IgG Fc antibody. This study revealed that isotype control and FITC-labeled anti-IgG Fc alone did not exhibit any cellular binding in the absence of the bispecific construct, whereas both HER2 × CD3 TDBA (**10a**) and HER2 × HER2 trivalent constructs (**10b**) did bind, as expected. Following this, the binding of the constructs to Jurkat cells was analyzed in an analogous manner. As expected, only the sample treated with the 2:1 HER2 × CD3 construct prior to staining showed an increase in fluorescence compared with untreated control, while the samples incubated with trivalent control (**10b**), isotype control, and FITC-labeled anti-IgG Fc antibodies alone did not exhibit any increase in fluorescence. Both results indicate that the synthesized constructs retained their binding capacity to the targeted cells even after the chemoenzymatic manipulations and purification steps.

Second, we tested for the presence of interferon-γ in the culture media to determine whether T cell binding was also accompanied by T cell activation (Figure 2B). This was done by ELISA, utilizing supernatants that were collected from the coculture or relevant control cultures 48 h after treatment (T cells were obtained from three healthy blood donors). Briefly, T cell/HCC1954 cocultures (effector:target or E/T ratio of 10:1), HCC1954 monocultures, or T cell monocultures were incubated with 5 nM of the bispecific construct **10a**, or any of the controls (i.e., untreated cells, trivalent HER2 construct **10b**, tras[LC]G_4_Y, MeTz-rbFab_CD3_, a 1:1 mixture of tras[LC]G_4_Y and MeTz-rbFab_CD3_). As shown in Figure 2B, interferon-γ secretion was observed only in the presence of 2:1 HER2 × CD3 TDBA **10a** in the T cell/HCC1954 coculture. Importantly, the absence of interferon-γ in the coculture treated with the 1:1 mixture of separate tras[LC]G_4_Y and MeTz-rbFab_CD3_ indicates that both building blocks need to be covalently linked in the same molecular construct in order to elicit the desired effector functions. Additionally, no interferon-γ production was observed in T cell monoculture treated with 2:1 HER2 × CD3 construct **10a**, which suggests that T cell activation would be primarily confined to the tumor microenvironment *in vivo*, and, as such, potentially indicates a reduced overstimulation of the immune system in the absence of tumor cells.^50^

Now that we confirmed that the constructs retained their affinity for their intended biological targets and that the T cells were properly activated to release interferon-γ, the stage was set to study the capability of each construct to induce T cell-mediated cell death of HER2^+^ HCC1954 tumor cells (Figure 2C). For this, monocultures of T cells or HCC1954 and coculture of T cell/HCC1954 cells (E/T ratio of 10:1) were incubated with 5 nM of each construct, and HCC1954 cell viability was measured 48 h post treatment. To our delight, >90% tumor cell death was observed in cocultures treated with the 2:1 HER2 × CD3 TDBA **10a**, while no significant reduction in cell viability was observed with any of the other constructs. The fact that a mixture of the non-linked components did not trigger T cell-mediated cell killing underscores that simultaneous binding of T cells and tumor cells by the same molecular construct is required for proper T cell activation against tumor cells.

Lastly, the IC_50_ of 2:1 HER2 × CD3 construct **10a** was determined using HCC1954 cells and T cells (E/T ratio of 10:1) (Figure 2D). Exposure of cocultures to increasing concentrations of our T cell-engaging bispecific antibody construct **10a** revealed very low IC_50_ values of 2.4–12.2 pM. In view of our observation that the tested sample contained 71% of the 2:1 bispecific construct—another major component, 24%, was identified as the inactive construct tras[LC]G_4_-TCO (**9**)—it can safely be concluded that the reported activities are conservative indications of the actual activity of the bispecific construct that was prepared. In comparison to the IC_50_ values obtained for our earlier described 2:2 HER2 × CD3 bispecific antibody constructs (which ranged from 1.3 pM to 26.7 pM), it appears that this new synthetic platform leads to 2:1 bispecific conjugates that are at least as active as the 2:2 bispecifics.^25^

## Conclusions

We developed a novel and rapid approach to access T cell-dependent 2:1 bispecific constructs by means of two subsequently performed IEDDA click reactions. Using our biogenic tyrosine SPOCQ-alkyne reaction, we first functionalized a genetically engineered therapeutic antibody with one TCO handle, to which a tetrazine-functionalized bioactive molecule was clicked in a second step. Analysis of the isolated fractions revealed that the constructs are of decent purity (>71%), containing only inactive starting materials as byproducts. The generated bispecific constructs retained the binding of both mAbs, activating the T cells to kill HER2^+^ HCC1954 cells, even at very low picomolar concentrations.^46^ With this additional approach to prepare 2:1 bispecific constructs, bispecific constructs can be generated with which the T cell response is elicited using only one T cell-binding epitope.

## Supporting Information Available

Synthetic chemistry experimental details; conjugation experiments and *in vitro* assays; ^1^H and ^13^C spectra; and full LC-MS spectra, including TIC trace and deconvoluted spectra (PDF)
