# Supplementary material for: Modular Semisynthetic Approach to Generate T Cell-Dependent Bispecific Constructs from Recombinant IgG1 Antibodies
Source: Bioconjug Chem. 2024 Sep 16;35(10):1524–31. doi: 10.1021/acs.bioconjchem.4c00309 (PMC11487494; doi:10.1021/acs.bioconjchem.4c00309)
Supplement: Supplementary file 1 — bc4c00309_si_001.pdf [file bc4c00309_si_001.pdf]

Supporting Information

for

A Modular Semi-Synthetic Approach to Generate T cell-Dependent Bispecific Constructs from Recombinant IgG1 Antibodies

Irene Shajan,[a] Léa N.C. Rochet,[b] Shannon R. Tracey,[c] Bianka Jackowska,[c] Rania Benazza,[d] Oscar Hernandez-Alba,[d] Sarah Cianférani,[d] Christopher J. Scott,[c] Floris L. van Delft,[a,e] Vijay Chudasama,[b] Bauke Albada[a,*]

[a]Laboratory of Organic Chemistry, Wageningen University & Research, Stippeneng 4, Wageningen, 6807 WE, the Netherlands

[b]Department of Chemistry, University College London, 20 Gordon St, London WC1H 0AJ, United Kingdom

[c]Patrick G Johnston Centre for Cancer Research, School of Medicine, Dentistry and Biomedical Sciences, Queen's University Belfast, 97 Lisburn Road, Belfast BT9 7BL, United Kingdom

[d]Laboratoire de Spectrométrie de Masse BioOrganique, Université de Strasbourg, CNRS, IPHC UMR 7178, 67000 F-Strasbourg, France

Infrastructure Nationale de Protéomique ProFI – FR2048, 67087 Strasbourg, France

[e]Synaffix BV – A Lonza company, Kloosterstraat 9, Oss 5349 AB, the Netherlands

*Corresponding author: bauke.albada@wur.nl

Table of Contents

	Page
General procedures	S3
Antibody modifications and bioconjugations	S4
Synthesis of linkers	S15
In vitro evaluation of the bispecific antibodies	S18
NMR spectra	S19
LC-MS data of the linkers	S27
Appendix	S29
Literature references	S30

General procedures

Starting materials, reagents, and solvents were purchased from commercial vendors and used as received unless stated otherwise. TCO-PEG$_3$-NH$_2$ was purchased from BroadPharm® (San Diego, CA, United States). *Endo*-bicyclo[6.1.0]non-4-yn-9-ol (BCN-OH) was provided by Synaffix BV (Oss, the Netherlands). Mushroom tyrosinase was purchased from Sigma-Aldrich. Trastuzumab (Herzuma) was obtained from the pharmacy. OKT3 antibody was purchased from BioLegend®. Tras[LC]G$_4$Y was ordered from Evitria AG, Switzerland. Reactions were monitored by thin-layer chromatography (TLC) using Merck aluminum sheets (Silica gel 60 F254). Organic solvents were removed under reduced pressure at 40 °C (at 33 °C for BCN-containing compounds). ^{1}H NMR and ^{13}C NMR spectra were recorded using a Brüker AV-400 (400 and 101 MHz, respectively) spectrometer in CDCl$_3$. Chemical shifts are given in ppm (δ) relative to the residual solvent peak or tetramethyl silane (0 ppm) as internal standard and coupling constants are given in Hz. High-resolution mass spectrometry (HRMS) analysis was performed with an Q-Exactive Focus Mass Spectrometer (Thermo Fisher), equipped with an electrospray ion source (ESI) in positive mode. Size exclusion chromatography was performed on AKTA pure system. Deconvoluted spectra were obtained using UniDec software.[51] SDS-PAGE gels were scanned in BIO-RAD ChemDoc XRS+ Imager.

General procedure for reducing SDS-PAGE, Coomassie staining and fluorescence detection

6% and 12% acrylamide gels were prepared according to BIO-RAD bulletin 6201 protocol. To 5 µg of the antibody in 5 µL PBS pH 7.4 was added 5 µL of 2× sample buffer and heated to 95 °C for 5 minutes. For reducing SDS-PAGE, 10% β-mercaptoethanol was added to the 2× sample buffer, prior to adding to the antibody sample. After loading the samples, the gel was run using a BIO-RAD Mini-PROTEAN Tetra Vertical Electrophoresis Cell at 120 volts until completion. The gel was stained using a 1 g/L Coomassie Brilliant Blue R-250 in 5:4:1 (v/v/v) methanol:water:acetic acid solution (30 minutes soak). The gel was subsequently destained using 5:4:1 (v/v/v) methanol:water:acetic acid for 30 minutes, after which it was further destained overnight using demineralized water.

General procedure for analytical RP-HPLC

Prior to RP-HPLC analysis, 40 µL of 12.5 mM DTT in 100 mM Tris.HCl pH 8 was added to IgG (10 µL, 1 mg/mL in PBS pH 7.4) and incubated for 15 minutes at 37 °C. The reaction was quenched by adding 49% acetonitrile, 49% water, 2% formic acid (50 µL). RP-HPLC analysis was performed on an Agilent 1290 series instrument. The sample (20 µL) was injected with 0.6 mL/min onto MAbPac RP 3.0 × 100 mm, 4 µm (Thermo Scientific) with a column temperature of 80 °C. A linear gradient was applied in 15 minutes from 25% to 40% acetonitrile with 0.1% FA and water with 0.1% FA. For the RP-HPLC analysis of intact mAb constructs, 5 µL of the sample in PBS pH 7.3 (conc. 0.1 mg/mL) was injected and a linear gradient was applied in 4 minutes from 15% to 50% acetonitrile with 0.1% TFA and water with 0.1% TFA.

Protein A purification of tras[LC]G$_4$Y

Trastuzumab with GGGGY on the light chains (tras[LC]G$_4$Y) was purified using a 5 mL HiTrap MabSelect SuRe™ protein A column on a BioLogic Duoflow™ chromatography system. The column was pre-equilibrated with 20 mM sodium phosphate, 150 mM NaC pH 7.2. Tras[LC]G$_4$Y in CHO media was applied to the column and washed with 10 column volumes of 20 mM sodium phosphate pH 7.0. Product was eluted with 5 column volumes of 0.1 M citric acid pH 3.0, obtained product fractions were diluted with 0.1 volume equivalents of 1.0 Tris buffer pH 9.0. The product fractions were concentrated and buffer exchanged to 50 mM potassium phosphate pH 7.3 containing 150 mM NaCl.

Size Exclusion Chromatography coupled to native Mass Spectrometry for the characterization of bispecific formats.

SEC-nMS measurements were performed on an Acquity UPLC-H-class system (Waters, Wilmslow, UK) comprising a quaternary solvent manager, a sample manager at 10°C, a column oven at room temperature and a TUV detector operating at 214 nm and 280 nm, coupled to an Orbitrap Exactive Plus EMR (Thermo Fisher Scientific, Bremen, Germany). Ten to twenty µg of mAbs and bi-specific formats were loaded on a MaxPeak Premier Protein SEC column (250Å, 1.7 µm, 4.6 x 150 mm) from Waters, Manchester, UK, using an isocratic gradient of 150 mM AcNOH$_4$ (pH 6.9) at a constant flowrate of 250 µL/min over 8 min. The EMR mass spectrometer was calibrated and tuned using a 2 g/L solution of cesium iodide in 2-propanol/water (50/50 v/v). Compounds were analyzed with a spray voltage of +4.0 kV. The in-source collision induced dissociation (CID) and the higher-energy collisional dissociation (HCD) were set to 150, and 10 V respectively. The trapping gas pressure was set to 7 a.u. (which corresponds to

an Ultra High Vacuum of 1.10-9 mbar). To improve the transmission of the high mass species, the voltages on the injection-, inter-, and bent-flatapoles were fixed to 8, 7, and 6 V, respectively. The orbitrap mass analyzer was operating at 17 500 resolution at 200 m/z with an automatic gain control (AGC target) fixed to 3*10^6, and a maximum injection time set to 300 ms. Acquisitions were performed in the 2 000-12 000 m/z range, and data processing was performed with BioPharma Finder v 3.2 (Thermo Fisher Scientific, Bremen, Germany).

Antibody bioconjugations

*Conjugation of tras[LC]G_4Y with bis-BCN (**1**)*

Tras[LC]G_4Y (3.14 µL, 31.864 mg/mL, 100 µg in PBS pH 5.5) was incubated with bis-BCN (**1**, 0.366 µL, 10 mg/mL in DMSO, 10 equiv.) and mushroom tyrosinase (8.6 µL, 10 mg/mL in phosphate buffer pH 6.0) at 4 °C. After overnight incubation, the product (**7**) was purified using protein A purification and buffer exchanged to PBS pH 5.5. RP-LC-MS analysis of the DTT digested product was performed as described above and indicated clean conversion and showed one major product at 9.3 mins (observed mass 48231 Da) corresponding to the expected intramolecularly crosslinked light chains.

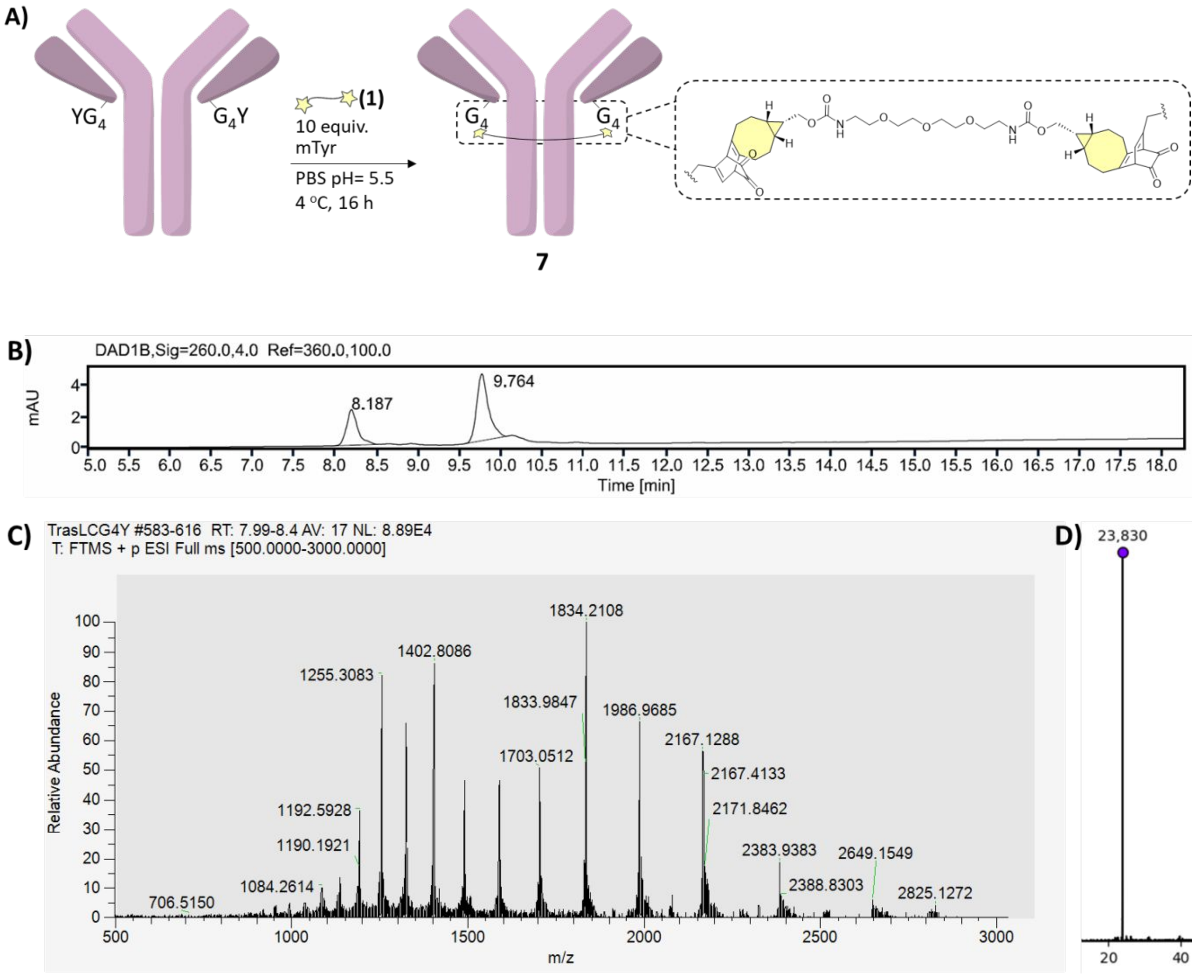

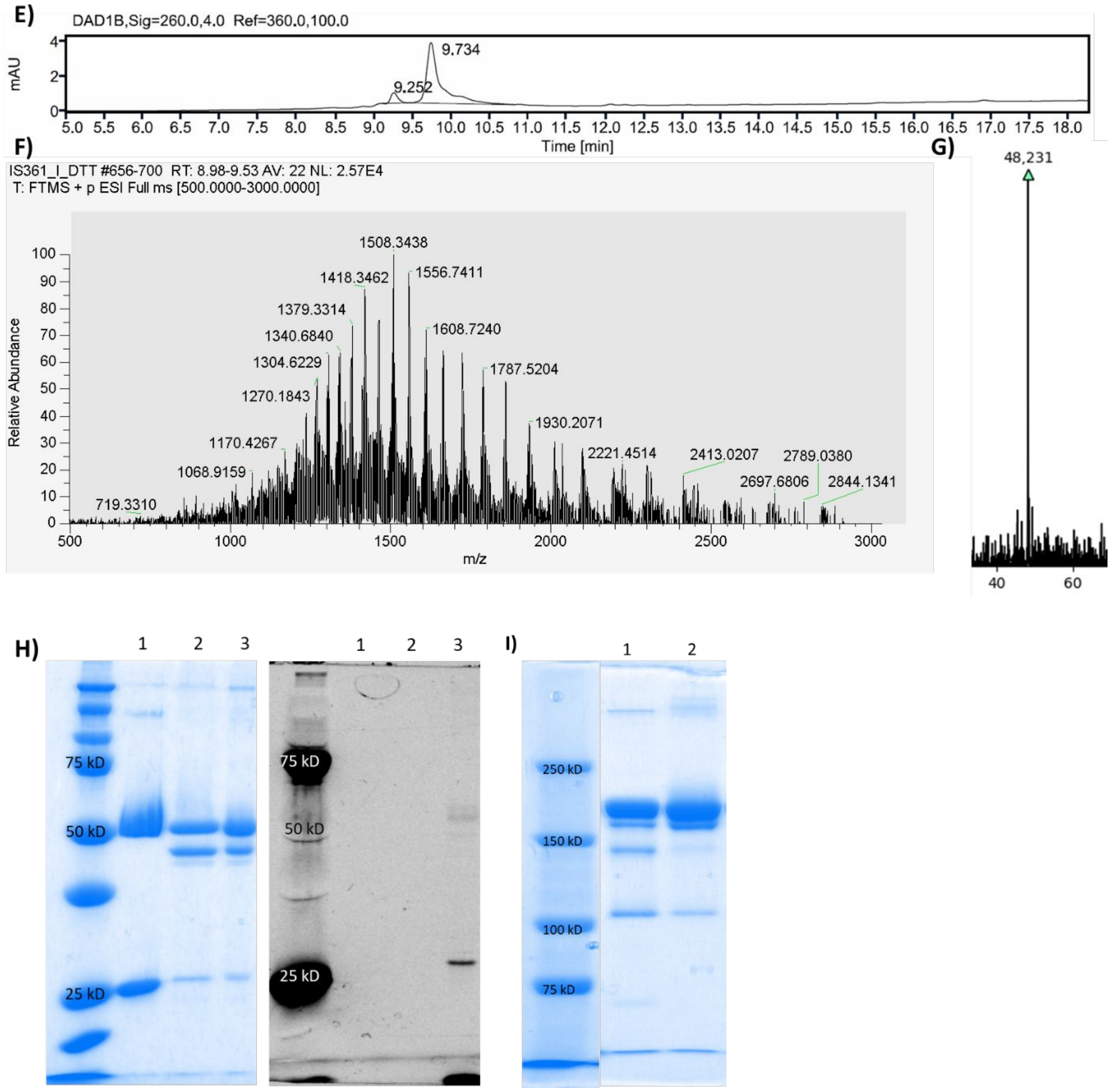

Fig. S1: A) Schematic representation of SPOCQ reaction between tras[LC]G$_4$Y and bis-BCN (**1**). B) HPLC trace of DTT digested tras[LC]G$_4$Y, with light chain at t_R 8.2 min and heavy chain at t_R 9.8 min. C) Mass spectrum of the light chain of tras[LC]G$_4$Y. D) Deconvoluted mass spectrum of light chain (the peak eluting at t_R = 8.2 min in panel B). E) HPLC trace of DTT digested tras[LC]G$_4$Y after SPOCQ with bis-BCN (**1**), with crosslinked light chains at t_R 9.3 min and heavy chain at t_R 9.7 min. F) Mass spectrum of the crosslinked light chains of tras[LC]G$_4$Y after SPOCQ with bis-BCN (**1**). G) Deconvoluted mass spectrum of the crosslinked light chains (the peak eluting at t_R = 9.3 min in panel E). H) Reducing SDS-PAGE of lane 1: tras[LC]G$_4$Y, lane 2: tras[LC]G$_4$Y after SPOCQ with bis-BCN (**1**) and lane 3: tras[LC]G$_4$Y after SPOCQ with bis-BCN (**1**) followed by IEDDA with tetrazine-TAMRA. In lane 2 and 3 the 25 kDa associated with the LC has mostly disappeared and a heavier band below the 50 kDa of the HC has appeared. Some of the LC was functionalized with a BCN, which became fluorescent after IEDDA with tetrazine-TAMRA (lane 3). I) Non-reducing SDS-PAGE of lane 1: tras[LC]G$_4$Y and lane 2: tras[LC]G$_4$Y after SPOCQ with bis-BCN (**1**).

*Conjugation of tras[LC]G$_4$Y with tri-BCN **2** to yield tras[LC]G$_4$-BCN (**8**)*

Tras[LC]G$_4$Y (3.14 µL, 31.864 mg/mL, 100 µg in PBS pH 5.5) was incubated with tri-BCN (**2**, 0.542 µL, 10 mg/mL in DMSO, 10 equiv.) and mushroom tyrosinase (8.6 µL, 10 mg/mL in phosphate buffer pH 6.0) at 4 °C. After overnight incubation, the product (**8**) was buffer exchanged to PBS pH 5.5 and concentrated. RP-LC-MS analysis of the DTT digested product was performed as described above and indicated clean conversion and showed one major product at 9.4 mins (observed mass 48493 Da) corresponding to the expected intramolecularly crosslinked light chains.

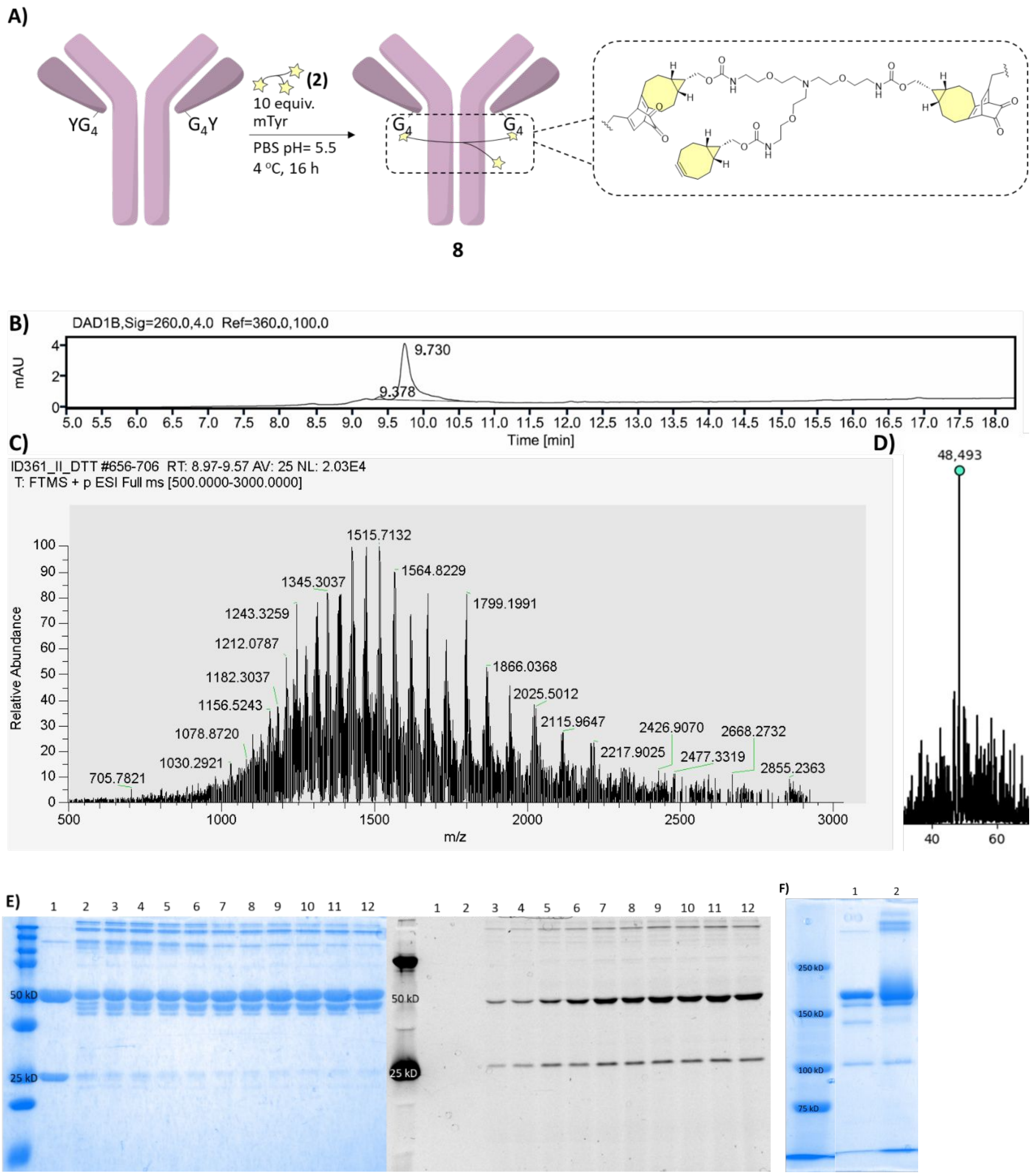

Fig. S2: A) Schematic representation of SPOCQ reaction between tras[LC]G$_4$Y and tri-BCN (**2**). B) HPLC trace of DTT digested tras[LC]G$_4$Y after SPOCQ with tri-BCN (**2**), with crosslinked light chains at t_R 9.4 min and heavy chain at t_R 9.7 min. C) Mass spectrum of the crosslinked light chains of tras[LC]G$_4$Y after SPOCQ with tri-BCN (**2**). D) Deconvoluted mass spectrum of the crosslinked light chains (the peak eluting at t_R = 9.4 min in panel B). E) Reducing SDS-PAGE of lane 1: tras[LC]G$_4$Y, lane 2: tras[LC]G$_4$Y+mTyr (control) and lanes 3–12: tras[LC]G$_4$Y after SPOCQ with different equivalence of tri-BCN (0.5, 1, 1.5, 2, 3, 4, 5, 6, 8, 10 equiv., respectively) and subsequent

IEDDA with MeTz-TAMRA. F) Non-reducing SDS-PAGE of lane 1: tras[LC]G_4Y and lane 2: tras[LC]G_4Y after SPOCQ with tri-BCN (**2**).

*Conjugation of tras[LC]G_4Y with BCN_2-TCO to yield tras[LC]G_4-TCO (**9**)*

Tras[LC]G_4Y (263.2 µL, 13.3 mg/mL, 3.5 mg in PBS pH 5.5) was incubated with BCN_2-TCO (**3**, 11.3 µL, 10 mg/mL in DMSO, 5 equiv.) and mushroom tyrosinase (301 µL, 10 mg/mL in phosphate buffer pH 6.0) at 4 °C. After overnight incubation, the product was purified using protein A purification and buffer exchanged to PBS pH 5.5. RP-LC-MS analysis of the DTT digested product was performed as described above and indicated clean conversion and showed one major product at 9.5 mins (observed mass 48648 Da) corresponding to the expected intramolecularly crosslinked light chains.

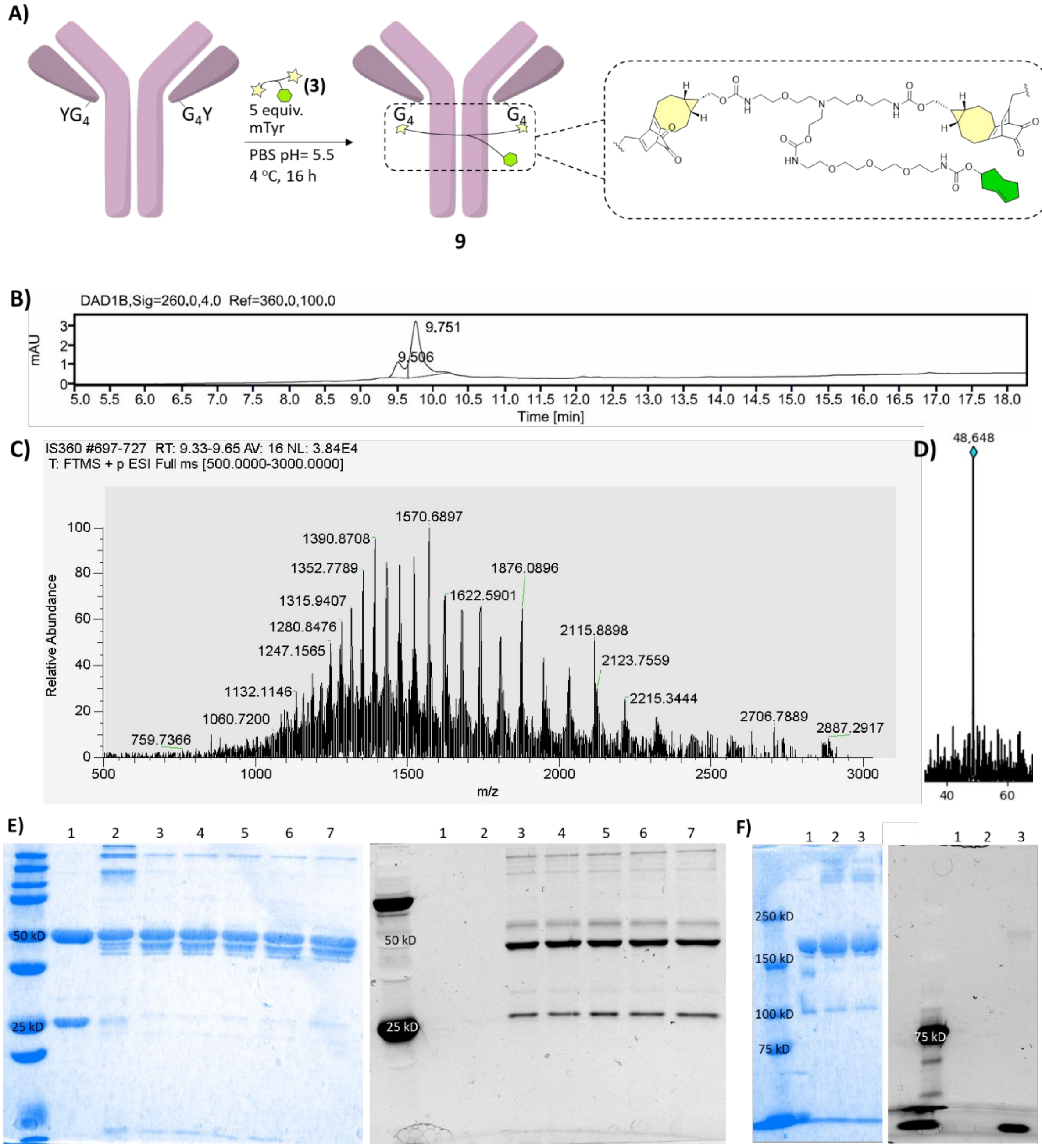

Fig. S3: A) Schematic representation of SPOCQ reaction between tras[LC]G_4Y and BCN_2-TCO (**3**). B) HPLC trace of DTT digested tras[LC]G_4Y after SPOCQ with BCN_2-TCO (**3**), with crosslinked light chains at t_R 9.5 min and heavy chain at t_R 9.8 min. C) Mass spectrum of the crosslinked light chains of tras[LC]G_4Y after SPOCQ with BCN_2-TCO (**3**). D) Deconvoluted mass spectrum of the crosslinked light chains (the peak eluting at t_R = 9.5 min in panel B). E)

Reducing SDS-PAGE of lane 1: tras[LC]G_4Y, lane 2: tras[LC]G_4Y+mTyr (control, showing crosslinking caused by nucleophilic residues that react with the quinone), lanes 3–7: tras[LC]G_4Y after SPOCQ with different equivalence of BCN_2-TCO (5, 10, 15, 20 and 30 equiv., respectively) and subsequent IEDDA with tetrazine-TAMRA (6 equiv.). F) Non-reducing SDS-PAGE of lane 1: tras[LC]G_4Y, lane 2: tras[LC]G_4-TCO (**9**), and lane 3: SPAAC of tras[LC]G_4-TCO with azido-TAMRA (which does not react with the TCO handle on the antibody).

*Digestion of mAb to Fab fragment (OKT3 and trastuzumab **5a/5b**)*

The buffer in which the mAb was stored was exchanged into sodium pepsin digest buffer (20 mM NaOAc, pH 3.1). Immobilized pepsin (732 µL) was washed 4 times with pepsin digest buffer and the mAb solution (1 mL, 107 µM) was added to this and the heterogeneous mixture was agitated (1100 rpm) constantly for 5 h at 37 °C. The resin was separated from the digest using a filter column and washed 3 times with papain digest buffer (50 mM sodium phosphate, 150 mM NaCl, 1 mM EDTA, pH 6.8). The obtained digest (containing the objected $F(ab')_2$ solution) was combined with the washes and the volume adjusted to 0.5 mL. Immobilized papain (1.22 mL, 0.25 mg/mL) was activated with 10 mM DTT (in papain digest buffer) with constant agitation (1100 rpm) for 90 min at 37 °C. The resin was washed 4 times with papain digest buffer (without DTT) and the 0.5 mL of $F(ab')_2$ solution was added. The mixture was incubated for 24 h at 37 °C under constant agitation (1100 rpm). The resin was separated from the digest using a filter column, washed 3 times with PBS and the digest combined with the washes. At the end, the buffer was exchanged completely for PBS (10 mM phosphate, 2.7 mM KCl, 137 mM NaCl, pH 7.4), and the volume adjusted to 0.5 mL.

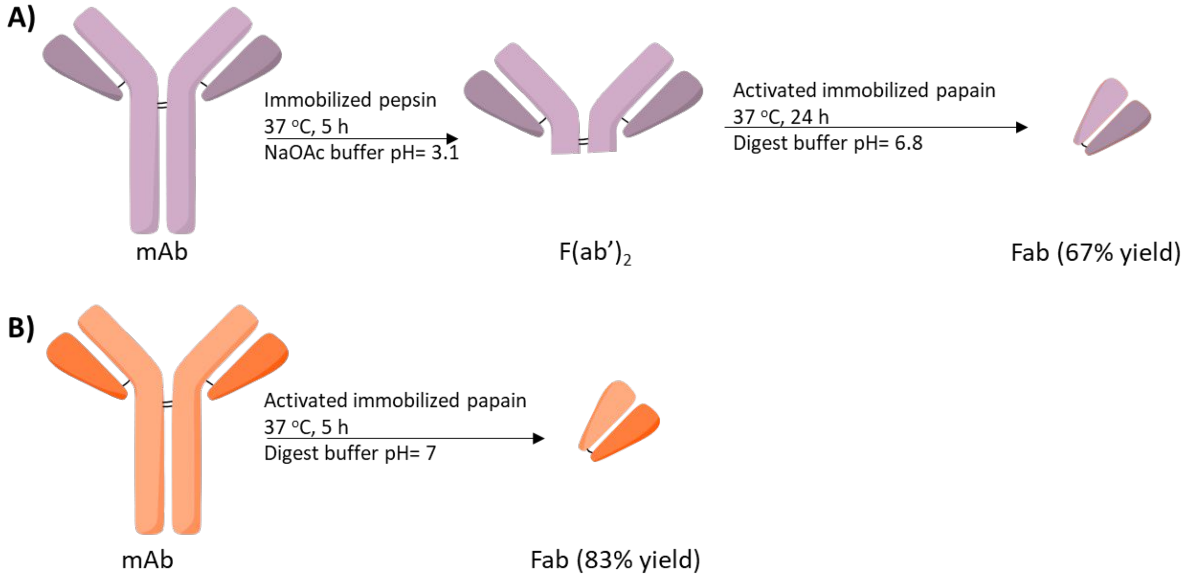

Fig. S4: Preparation of the A) Fab_{HER2} (**5b**) and B) Fab_{CD3} (**5a**) fragment from their respective parent antibodies.

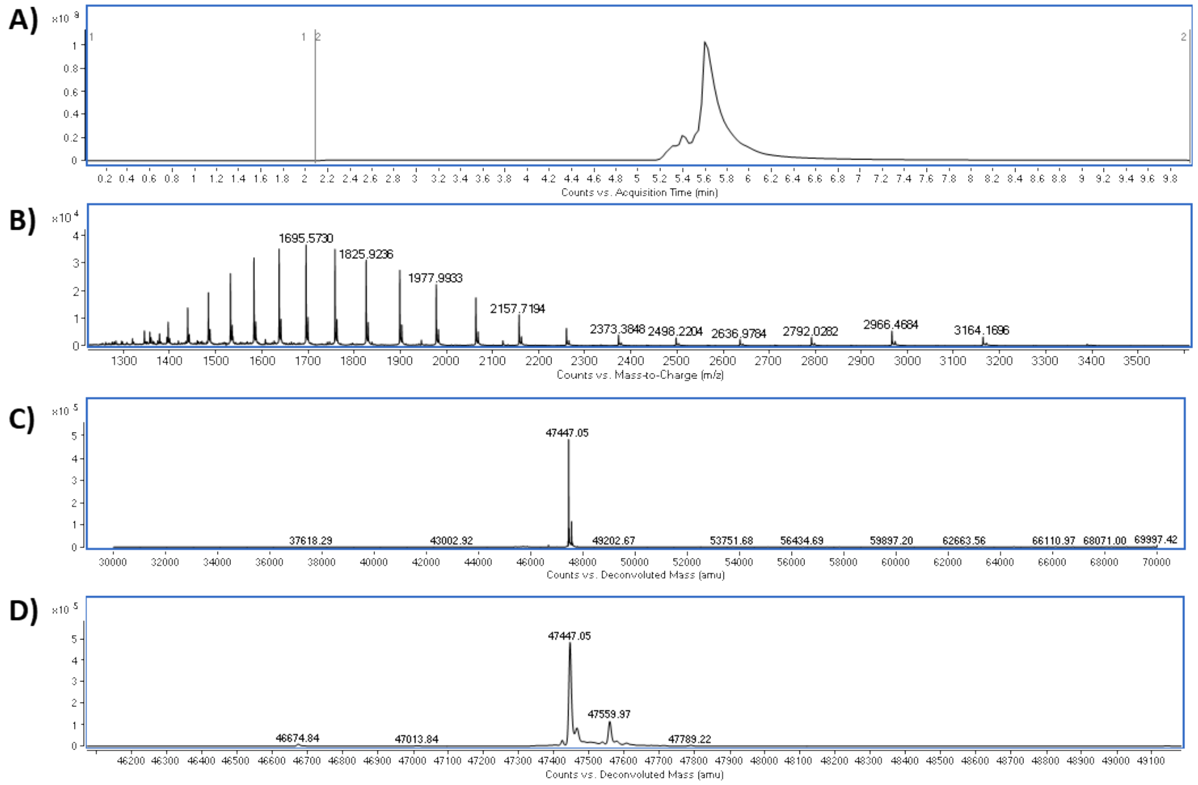

Fig. S5: A) MS chromatogram, B) mass spectrum, C) deconvoluted spectrum, and D) zoomed deconvoluted spectrum of Fab_{CD3} (**5a**). Expected masses: 47447, 47560 Da (latter value likely corresponds to an additional Leu in the primary sequence).

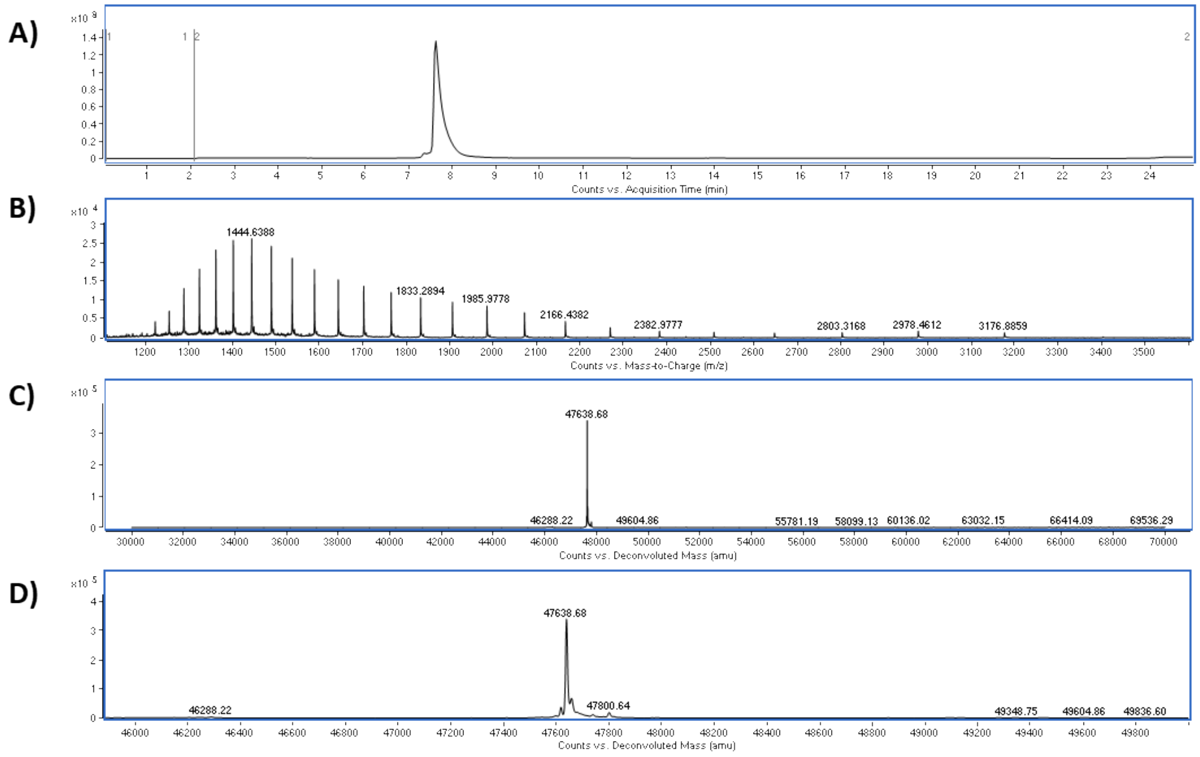

Fig. S6: A) MS chromatogram, B) mass spectrum, C) deconvoluted spectrum, and D) zoomed deconvoluted spectrum of Fab_{HER2} (**5a**). Expected mass: 47639 Da.

*Reduction of Fab fragment and subsequent Fab rebridging with tetrazine-dibromopyridazinedione (**4**)*

A solution of 20 mM TCEP was prepared by dissolving TCEP-HCl (15 mg) in 5 × BBS (2.6 mL). Fab (10 µM, 200 µL) was prepared in BBS and 5 × BBS was added (100 µL), followed by addition of 20–60 equiv. of TCEP (20 mM in 5 × BBS, 0.5–6 µL). The mixture was incubated for 120 min at 37 °C under constant agitation (300 rpm). The buffer was then exchanged for BBS to remove excess TCEP (Viva + Zeba spin). TCEP-HCl equivalents used for reduction: 60 eq. for Fab_{CD3} and 20 equiv. for Fab_{HER2}. For rebridging, to a solution of reduced Fab (100 µL, 20 µM) in BBS was added 5–20 equivalents of pyridazinedione Br_2PD-MeTz (**4**) (0.5–2 µL, 20 mM in DMSO) and the mixture incubated at 37 °C with constant agitation (300 rpm) over 2 h. The buffer was then exchanged for BBS to remove excess PD (Viva + Zeba spin). The purity of the sample was assessed by non-reducing SDS-PAGE and (HR)-LC-MS.

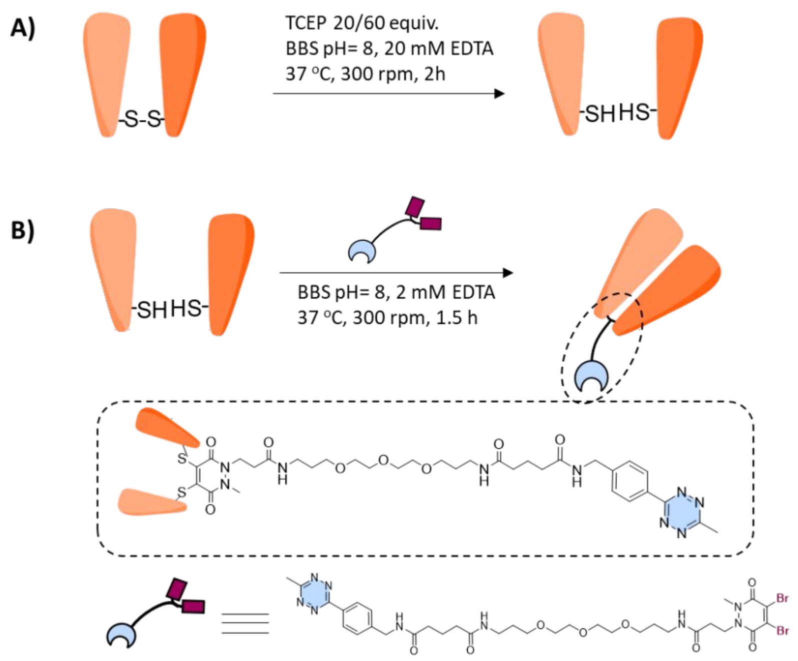

Fig. S7: A) Reduction of the Fab fragment, and B) Fab re-bridging using tetrazine-dibromopyridazinedione (**4**).

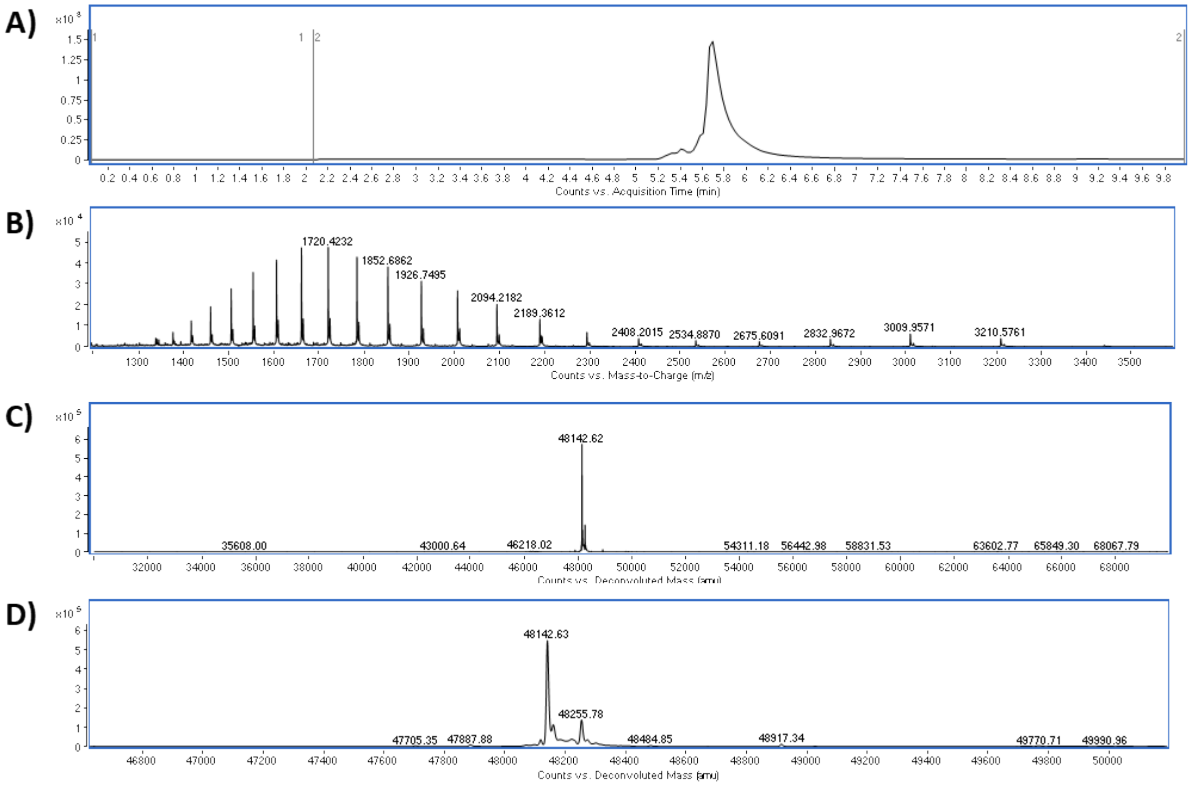

Fig. S8: A) MS chromatogram, B) mass spectrum, C) deconvoluted spectra and D) zoomed deconvoluted spectra of MeTz-rbFab$_{CD3}$ (**6a**). Expected masses: 48142, 48255 Da. *The mass 48255 Da is a side product from the previous digestion step.

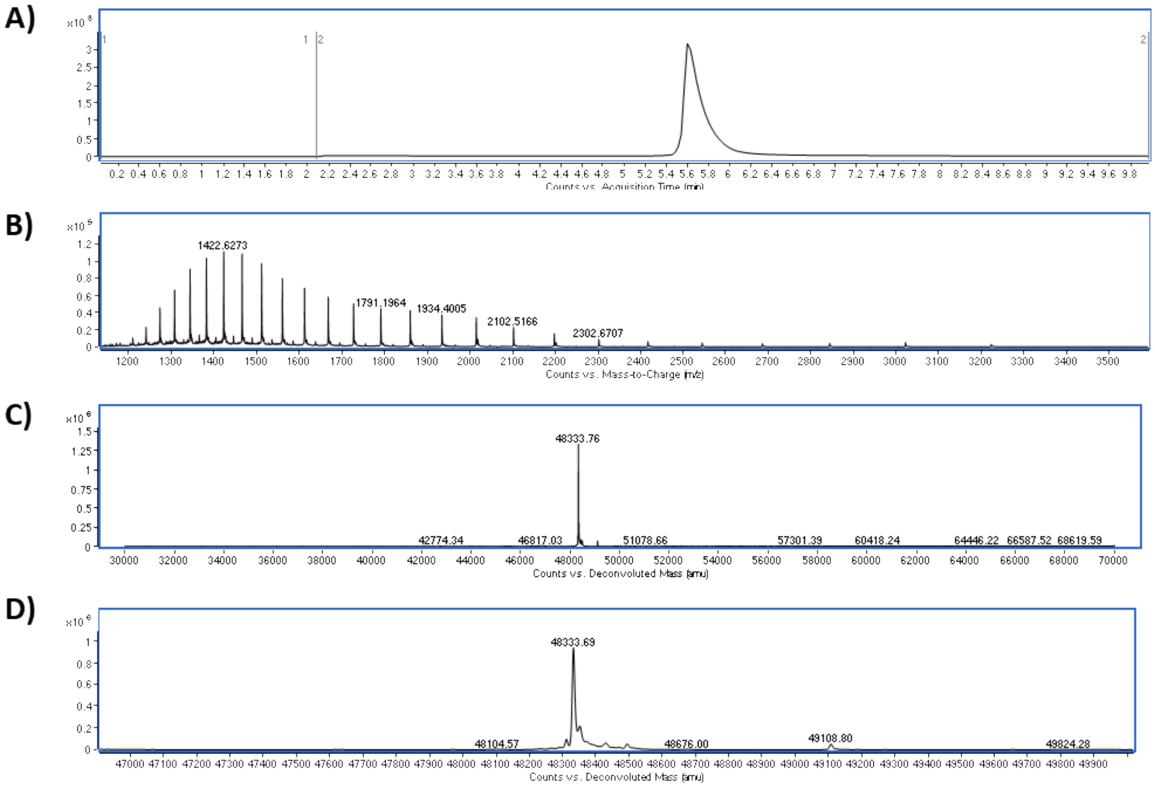

Fig. S9: A) MS chromatogram, B) mass spectrum, C) deconvoluted spectra and D) zoomed deconvoluted spectra of MeTz-rbFab$_{HER2}$ (**6b**). Expected masses: 48334 Da.

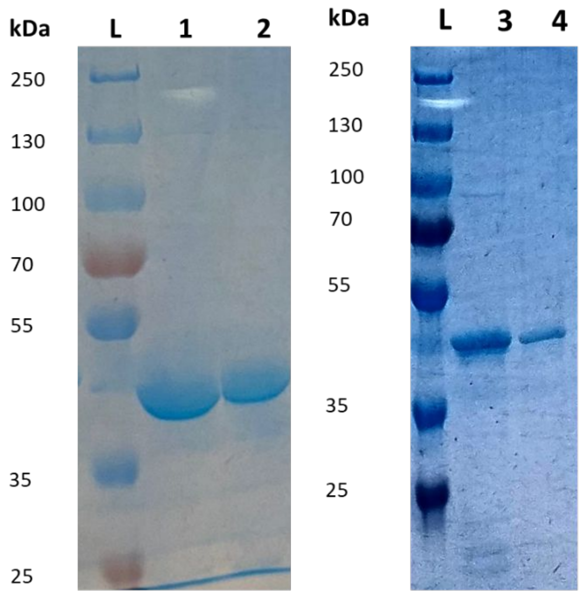

Fig. S10: 12% non-reducing SDS-PAGE gel of digested Fabs and rebridged Fabs. **L:** ladder, lane 1: Fab_{CD3} (**5a**); lane 2: MeTz-$rbFab_{CD3}$ (**6a**); lane 3: Fab_{HER2} (**5b**); lane 4: MeTz-$rbFab_{HER2}$ (**6b**).

*Generation of 2:1 HER2xCD3 bispecific antibody (**10a**)*

Tras[LC]G_4-TCO (91.4 µL, 27.4 mg/mL, 2.5 mg) was diluted with 700 µL PBS pH 5.5 and incubated with MeTz-rbFab$_{CD3}$ (13.1 µL, 55.4 mg/mL, 0.9 equiv.) at 4 °C for 2 h. The product was purified using protein A purification followed by size-exclusion chromatography, and analyzed using non-reducing SDS-PAGE and native SEC-MS.

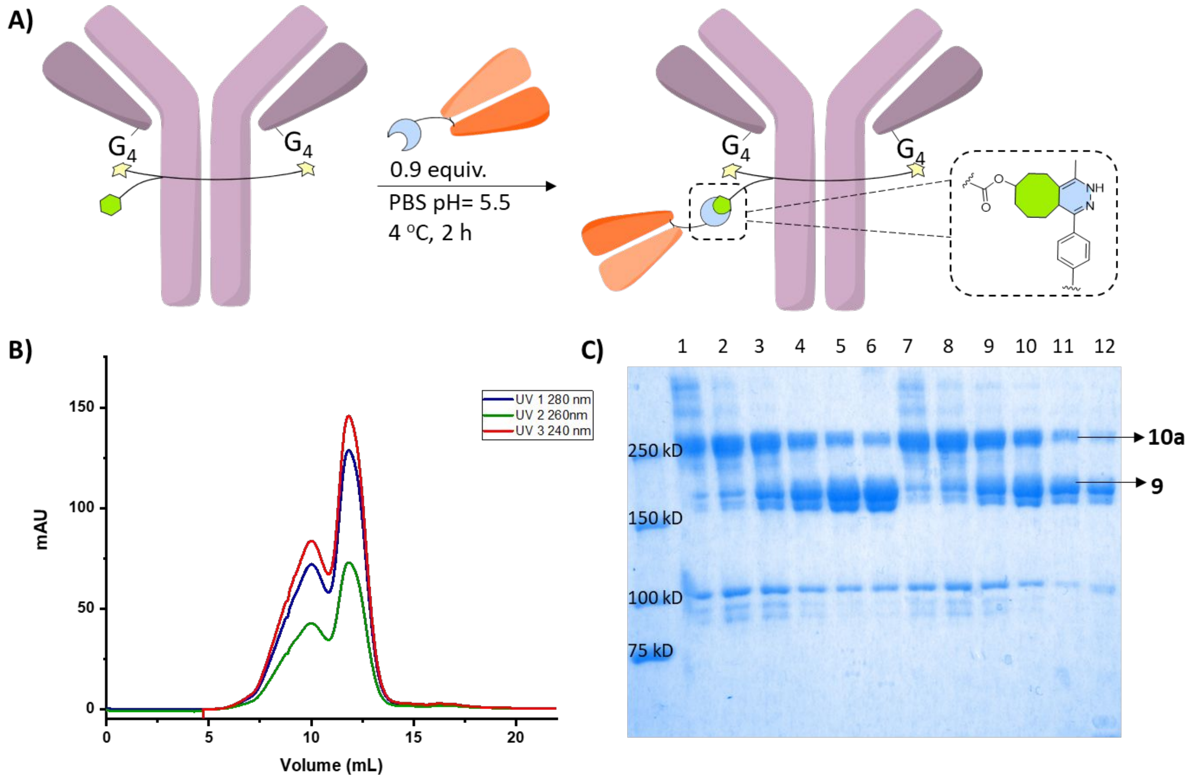

Fig. S11: A) Generation of 2:1 HER2×CD3 bispecific antibody via TCO-tetrazine IEDDA reaction, B) SEC purification of the reaction mixture, and C) 6% non-reducing SDS-PAGE of the SEC fractions: lane 1–12 corresponds to different fractions collected during SEC purification (sample was purified in two batches: lanes 1–6 are from the fractions that were collected after the first injection and lanes 7–12 are from the fractions that were collected after the second injection).

*<u>Generation of 2:1 HER2xHER2 bispecific antibody (**10b**)</u>*

Tras[LC]G_4-TCO (91.4 µL, 27.4 mg/mL) was diluted with 700 µL PBS pH 5.5 and incubated with tetrazine-trastuzumab Fab (14.2 µL, 51.1 mg/mL, 0.9 equiv.) at 4 °C for 2 h. The product was purified using protein A purification followed by size-exclusion chromatography, and analyzed using non-reducing SDS-PAGE and native SEC-MS.

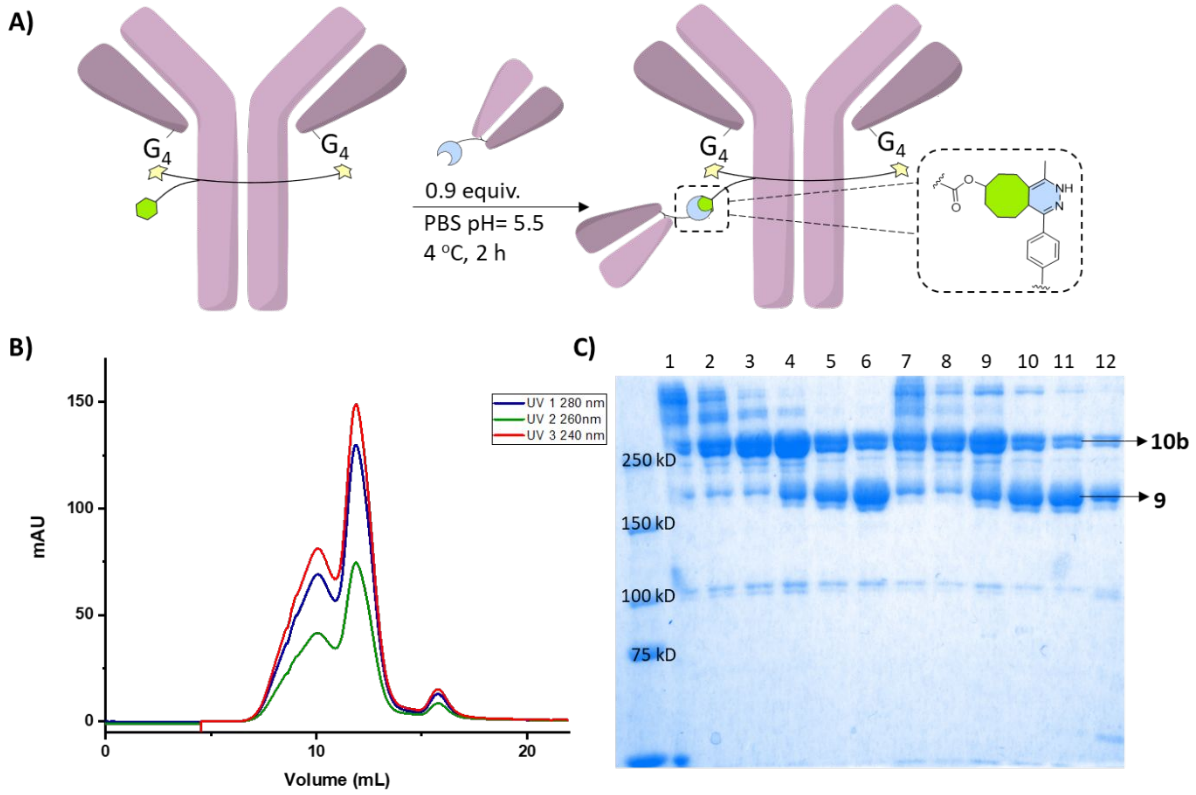

Fig. S12: A) Generation of 2:1 HER2×HER2 bispecific antibody via TCO-tetrazine IEDDA reaction, B) SEC purification of the reaction mixture, and C) 6% non-reducing SDS-PAGE of the SEC fractions: lane 1–12 corresponds to different fractions collected during SEC purification (sample was purified in two batches: lanes 1–6 are from the fractions that were collected after the first injection and lanes 7–12 are from the fractions that were collected after the second injection).

.

Synthesis of Linkers

*Synthesis of bis-BCN(PEG$_3$) (**1**)*

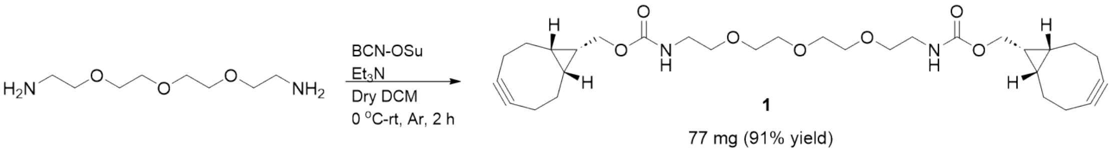

Fig. S13: Synthesis scheme for bis-BCN(PEG$_3$) (**1**).

BCN-OSu

To a solution of BCN-OH (250 mg) in 6 mL ACN stirred on ice were added disuccinimidyl carbonate (853 mg, 2 equiv.) and freshly distilled triethylamine (695.8 μL, 3 equiv.). After the mixture was stirred for 2 h at room temperature in dark, it was concentrated *in vacuo* at 33 °C. The residue was purified by silica gel column chromatography (25 → 50% (v/v) gradient of EtOAc in hexane) to afford the product as a white solid (273.3 mg, 57% yield). ^{1}H NMR (400 MHz, $CDCl_3$): δ 4.38 (d, 2H, *J* = 8 Hz), 2.77 (s, 4H), 2.29–2.13 (6H, m), 1.54–1.39 (3H, m), 1.04–0.94 (2H, m). ^{13}C NMR (101 MHz, $CDCl_3$): δ 168.7, 151.6, 98.7, 70.3, 29.0, 25.5, 21.5, 20.7, 17.2.

Bis-BCN(PEG$_3$) (**1**)

To a solution of 1,11-diamino-3,6,9-trioxaundecane (30 mg) in 5 mL dry DCM stirred at 0 °C, were added 47.8 μL freshly distilled triethylamine followed by BCN-OSu (90.9 mg, 2 equiv.). After stirring at room temperature for 2 h under argon atmosphere, the reaction was quenched with 10 mL saturated NH_4Cl and extracted with DCM (3 × 10 mL). The organic layer was washed with saturated $NaHCO_3$ (10 mL), dried over Na_2SO_4 and after filtration the solvent was evaporated *in vacuo* at 33 °C. The obtained residue was then purified via silica gel chromatography (3% MeOH/DCM) to afford the target product as a colorless oil (77 mg, 91% yield). ^{1}H NMR (400 MHz, $CDCl_3$): δ 5.23 (br s, 2H), 4.16 (d, *J* = 8 Hz, 4H), 3.63 (br s, 8H), 3.58–3.55 (t, *J* = 8 Hz, 4H), 3.39–3.35 (m, 4H), 2.33–2.18 (m, 12H), 1.66–1.53 (m, 5H), 1.40–1.31 (m, 2H), 0.98–0.89 (m, 4H). ^{13}C NMR (101 MHz, $CDCl_3$): δ 156.8, 98.8, 70.5, 70.3, 70.1, 62.7, 40.8, 29.1, 21.4, 20.1, 17.8. ESI-MS (observed m/z): 545.3219 Da.

*Synthesis of Tri-BCN (**2**)*

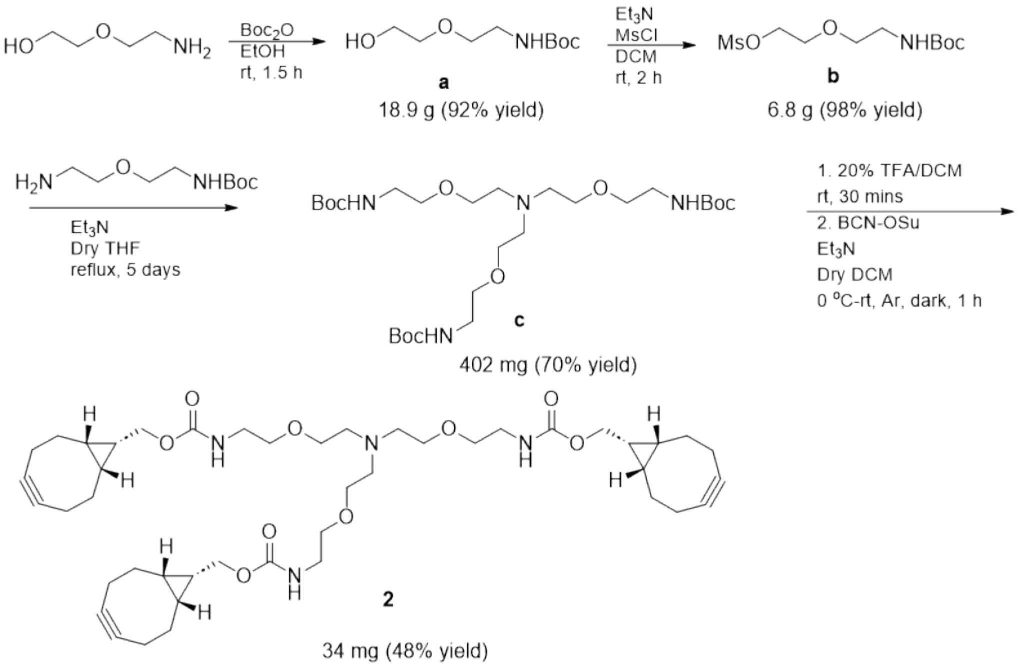

Fig. S14: Synthesis scheme for tri-BCN (**2**)

tert-Butyl (2-(2-hydroxyethoxy)ethyl)carbamate (**a**)[52]

2-(2-aminoethoxy)ethan-1-ol (10.5 g, 99.87 mmol) was dissolved in 100 mL EtOH and cooled to 0 °C in an ice-water bath. Di-*tert*-butyl dicarbonate (21.8 g, 99.87 mmol, 1 equiv.) was added portion-wise to the solution before purging the reaction vessel with argon. The flask with the reaction mixture was removed from the ice-water bath and stirred for 2 hours at room temperature. Upon completion, the solvent was removed under vacuum. The resulting colorless oil was dissolved in DCM (50 mL), and the organic phase was subsequently washed with water (50 mL) and brine (50 mL), after which it was dried over Na_2SO_4, filtered, and the filtrate concentrated *in vacuo* to afford the intended product (18.9 g, 92% yield) as a colorless oil. ^{1}H NMR (400 MHz, $CDCl_3$) δ 3.66 (t, *J* = 4 Hz, 2H), 3.51–3.47 (m, 4H), 3.25 (t, *J* = 4 Hz, 1H), 1.37 (s, 9H).

2-(2-((*tert*-butoxycarbonyl)amino)ethoxy)ethylmethanesulfonate (**b**)

Mesylate (**b**) was synthesized as described for compound 56 in WO2022031772A1. In short, to a solution of **3** (5 g) in 35 mL dry DCM at 0 °C was added freshly distilled triethylamine (7.5 mL, 2.2 equiv.) followed by mesyl chloride (2.07 mL, 1.1 equiv.). The ice bath was removed and the reaction mixture was stirred at rt for 2 h, after which it was diluted with DCM (200 mL), then washed with water (100 mL) and brine (100 mL). The organic layer was dried over Na_2SO_4, filtered, and the filtrate was concentrated *in vacuo* to afford the product (6.8 g, 98% yield) as a yellow oil. ^{1}H NMR (400 MHz, $CDCl_3$) δ 4.85 (br s, 1H), 4.31–4.29 (m, 2H), 3.68–3.65 (m, 2H), 3.49 (t, *J* = 8 Hz, 2H), 3.26 (t, *J* = 4 Hz, 2H), 3.00 (s, 3H), 1.38 (s, 9H).

Tri-*tert*-butyl carbamate (**c**)

To a solution of *tert*-butyl (2-(2-aminoethoxy)ethyl)carbamate (200 mg) in 3 mL dry THF were added triethylamine (545.8 µL, 4 equiv.) and mesylate **b** (832.3 mg, 3 equiv.). The reaction mixture was refluxed for 5 days and then concentrated *in vacuo*. The crude mixture was purified w/o workup via silica gel chromatography (100% EtOAc) to afford as yellow oil (402 mg, 70% yield). ^{1}H NMR (400 MHz, $CDCl_3$) δ 5.24 (br s, 3H), 3.55–3.48 (m, 12H), 3.30–3.26 (m, 6H), 2.78–2.75 (m, 6H), 1.43 (s, 27H); ^{13}C NMR (101 MHz, $CDCl_3$) δ 156.2, 79.3, 70.5, 70.2, 54.9, 40.4, 28.6.

Tri-BCN (**2**)

Boc deprotection of compound **c** was performed using 20% TFA in DCM (4 mL) at room temperature. After 30 minutes, the reaction mixture was concentrated *in vacuo* and the residues was co-evaporated thrice with toluene. To 54.3 mg of deprotected triamine dissolved in 5 mL dry DCM at 0 °C was added distilled triethylamine (73.2 µL, 6 equiv.) and BCN-OSu (126 mg, 4.9 equiv.). After stirring at room temperature for 2 h, the reaction mixture was diluted with 30 mL DCM and washed with 10 mL saturated NH_4Cl. The organic layer was washed with 10 mL brine, dried over Na_2SO_4, and the solvent of the resulting filtrate was evaporated at reduced pressure at 33 °C. The residue was then purified via silica gel chromatography (4% MeOH-DCM) to obtain the trifunctional BCN-linker **2** as product (33.9 mg, 48% yield). ^{1}H NMR (400 MHz, $CDCl_3$) δ 4.15 (d, *J* = 8 Hz, 6H), 3.97 (br s, 3H), 3.58–3.52 (br s, 13H), 3.37 (br s, 8H), 2.84–2.75 (m, 3H), 2.22–2.18 (m, 17H), 1.63–1.52 (m, 6H), 1.41–1.30 (m, 4H), 0.98–0.90 (m, 6H) ; ^{13}C NMR (101 MHz, $CDCl_3$) δ 156.9, 98.8, 70.2, 65.1, 62.7, 55.7, 40.4, 29.1, 21.4, 20.1, 17.8. ESI-MS (observed *m*/*z*): 807.4901 Da.

*Synthesis of BCN$_2$-TCO (**3**)*

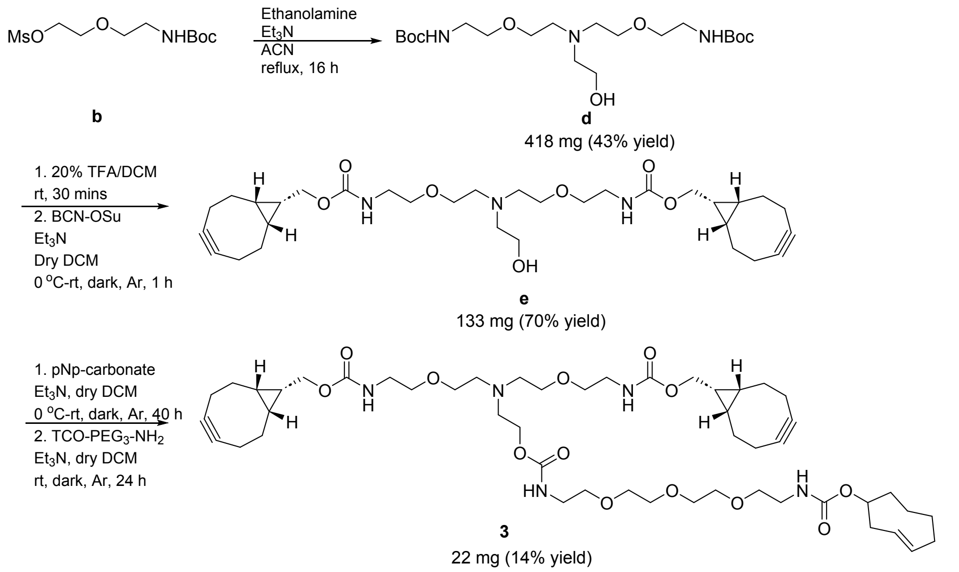

Fig. S15: Synthesis scheme for BCN$_2$-TCO (**3**)

di-*tert*-butyl alcohol (**d**)

To a solution of aminoethanol (136.5 mg) in 10 mL dry ACN were added triethylamine (311.5 µL, 1 equiv.) and mesylate **b** (Fig. S14) (1.9 g, 3 equiv.). The reaction mixture was heated to reflux for 16 hours and then concentrated *in vacuo*. The residue was dissolved in EtOAc and washed with saturated $NaHCO_3$. The organic layer was dried over Na_2SO_4 and after filtration the solvent of the filtrate was evaporated at reduced pressure. The crude mixture was purified using silica gel chromatography (1–3% MeOH/DCM) to afford as yellow oil (418 mg, 43% yield). ^{1}H NMR (400 MHz, $CDCl_3$) δ 5.23 (br s, 2H), 3.53–3.47 (m, 6H), 3.44 (t, *J* = 4 Hz, 4H), 3.27–3.22 (m, 4H), 2.80–2.73 (m, 4H), 2.71 (m, 2H), 1.38 (s, 18H); ^{13}C NMR (101 MHz, $CDCl_3$) δ 156.1, 79.2, 77.2, 70.1, 69.2, 59.0, 56.8, 54.5, 40.4, 28.4.

bis-BCN-alcohol (**e**)

Boc deprotection of 100 mg of **d** was done in 20% TFA-DCM mixture (4 mL) at room temperature. After 90 minutes, the reaction mixture was evaporated and then co-evaporated thrice with toluene. To 106 mg of deprotected analogue of **d** in 2.5 mL dry DCM at 0 °C were added distilled triethylamine (127.5 µL, 4 equiv.) and BCN-OSu (139.9 mg, 2.1 equiv.). After stirring at room temperature for 1 h, the reaction mixture was concentrated at reduced pressure. The residue was then purified via silica gel chromatography (2–10% MeOH/DCM) to obtain the intended product **e** (77 mg, 57% yield). ^{1}H NMR (400 MHz, $CDCl_3$) δ 5.51 (br s, 2H), 4.09 (d, *J* = 8 Hz, 4H), 3.57–3.51 (m, 6H), 3.48–3.45 (t, *J* = 4 Hz, 5H), 3.32–3.28 (m, 4H), 2.88–2.71 (m, 6H), 2.27–2.10 (m, 11H), 1.57–1.46 (m, 4H), 1.36–1.25 (m, 2H), 0.93–0.83 (m, 4H); ^{13}C NMR (101 MHz, $CDCl_3$) δ 156.9, 98.8, 70.1, 68.9, 62.7, 58.7, 54.5, 40.8, 39.7, 29.1, 21.4, 20.1, 17.8.

BCN$_2$-TCO (**3**)

To a solution of **e** (90 mg) in dry DCM (1 mL) at 0 °C, were added bis(4-nitrophenyl) carbonate (51.1 mg, 1.2 equiv.) and distilled triethylamine (70.4 µL, 3 equiv.). The reaction mixture was stirred in dark (0 °C→rt) for 40 h after which the mixture was treated with a solution of TCO-PEG$_3$-amine (126.8 mg, 1.1 equiv.) and distilled triethylamine (70.4 µL, 3 equiv.) in dry DCM (100 µL). After 24 h, it was concentrated *in vacuo* at 33 °C. The residue was purified by silica gel chromatography (5% MeOH/EtOAc) to obtain the target product **3** as an off-white film (22.1 mg, 13.7% yield). ^{1}H NMR (400 MHz, $CDCl_3$) δ 5.60–5.49 (m, 3H), 4.33 (br s, 1H), 4.15 (d, *J* = 8 Hz, 4H), 3.63 (br s, 9H), 3.58–3.53 (m, 9H), 3.37 (br s, 7H), 2.81 (br s, 4H), 2.37–2.18 (m, 15H), 2.06–1.88 (m, 5H), 1.74–1.68 (m, 2H), 1.62–1.52 (m, 5H), 1.42–1.19 (m, 10H), 0.98–0.82 (m, 6H); ^{13}C NMR (100 MHz, $CDCl_3$) δ 157.2, 156.6, 135.1, 133.1, 99.0, 80.7, 70.5, 62.9, 54.4, 41.3, 40.8, 38.8, 34.4, 32.7, 32.1, 31.1, 29.8, 29.2, 22.8, 21.6, 20.3, 17.9, 14.3. ESI-MS (observed m/z): 958.5752 Da.

Dibromopyridazinedione-tetrazine (**4**) was synthesized as described by Shajan *et al.*[25]

In vitro analyses

Cell culture

Jurkat cells and HCC1954 cells were cultured in RPMI 1640 supplemented with 10% v/v FBS, 50 units/mL penicillin and 50 µg/mL streptomycin. Primary human T cells were isolated from the buffy coat fractions of anonymized healthy blood donors after obtaining ethical approval from the Queen's University Belfast Research Ethics Committee/Northern Ireland Blood Transfusion Service. Purified T cells were cultured in RPMI 1640 supplemented with 10% v/v FBS, 50 units/mL penicillin and 50 µg/mL streptomycin. All cells were maintained in 5% CO_2 at 37 °C in a humidified incubator.

Flow cytometry

A total of 2.5 × 10^5 HCC1954 cells or Jurkat cells were resuspended in 1 mL media containing 2.5 nM of the bispecific antibody or control and incubated for 1 h at 4 °C. Cells were then washed by centrifugation at 600g for 5 min at 4 °C and resuspended in 1 mL FACS buffer (5% v/v FBS in PBS) and centrifuged again. The cells were resuspended in 100 µL FACS buffer containing 1.25 µg/mL FITC anti-human IgG Fc antibody. Following incubation for 30 mins at 4 °C in dark, cells were washed twice in FACS buffer by centrifugation at 600g for 5 mins at 4 °C, resuspended in PBS buffer and fluorescence was assessed on a BD Accuri C6 Plus flow cytometer.

T cell purification

Primary human T cells were isolated from the buffy coat fractions of anonymised healthy blood donors after obtaining ethical approval from the Queen's University Belfast Research Ethics Committee (Study Reference 2019/11 - Title: Evaluation of novel therapeutic compounds on human blood cells extracted from buffy coat). Buffy coats were gifted to Queens University Belfast as research material from Northern Ireland Blood Transfusion Service (MTA Number RG-475). The peripheral blood mononuclear cell (PBMC) fraction was transferred to a fresh tube, resuspended in 50 mL HBSS and centrifuged at 300g for 10 min at room temperature. To facilitate platelet removal, this HBSS resuspension-centrifugation step was repeated twice at a lower speed of 200g. From this, T cells were then purified from the PBMC fraction by negative selection using the EasySep™ Human T Cell Isolation Kit (STEMCELL Technologies) in accordance with the manufacturer's instructions.

T-cell/HCC1954 cell coculture

HCC1954 cells were seeded at 5 × 10^3 per well in 96-well plates and left to adhere overnight. Purified T cells were then added to appropriate wells at an effector: target (E:T) ratio of 10:1 followed by 5 nM treatment. After 48 h, culture supernatants were collected and centrifuged to remove cells. Cell-free supernatants were stored at –80 °C in preparation for downstream ELISA analysis. Microplate wells were then used to assess the viability of remaining adherent HCC1954 cells by CellTiter-Glo® assay in accordance with the manufacturer's instructions.

ELISA

Cell-free culture supernatants were assayed using the human IFN-γ DuoSet ELISA (R&D Systems) in accordance with the manufacturer's instructions.

Dose-response study

HCC1954 cells were seeded at 5 × 10^3 per well in 96-well plates and left to adhere overnight. Purified T cells were then added to appropriate wells at an effector:target (E:T) ratio of 10:1 followed by the addition of varying concentrations (0.01289 pM to 5 nM) of the treatment. After 48 h, culture supernatants were removed and the microplate wells were used to assess the viability of remaining adherent HCC1954 cells by CellTiter-Glo® assay in accordance with the manufacturer's instructions.

Data analysis

FlowJo software (version 10.8.1) was used to construct histograms in Figure 4/A. GraphPad Prism software (version 9.3.1) was used to graph data.

NMR spectra

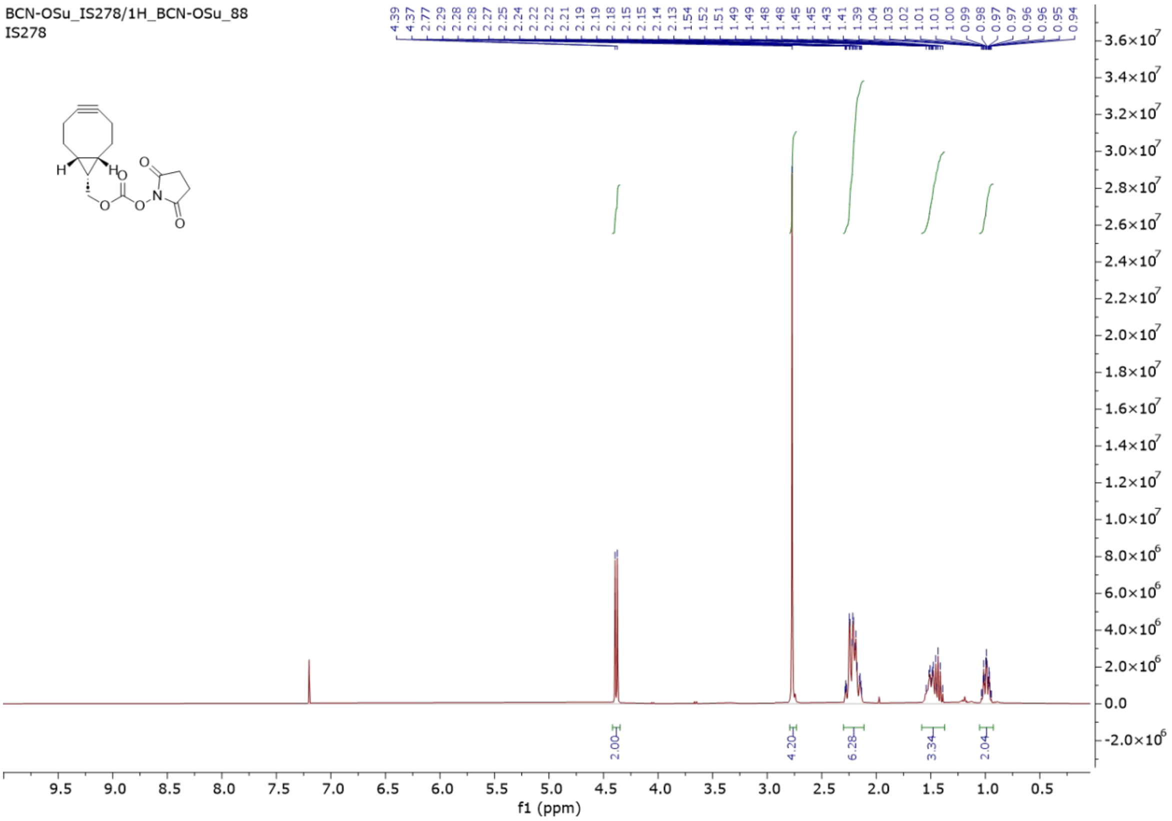

Fig. S17: ^{1}H NMR spectrum of BCN-OSu.

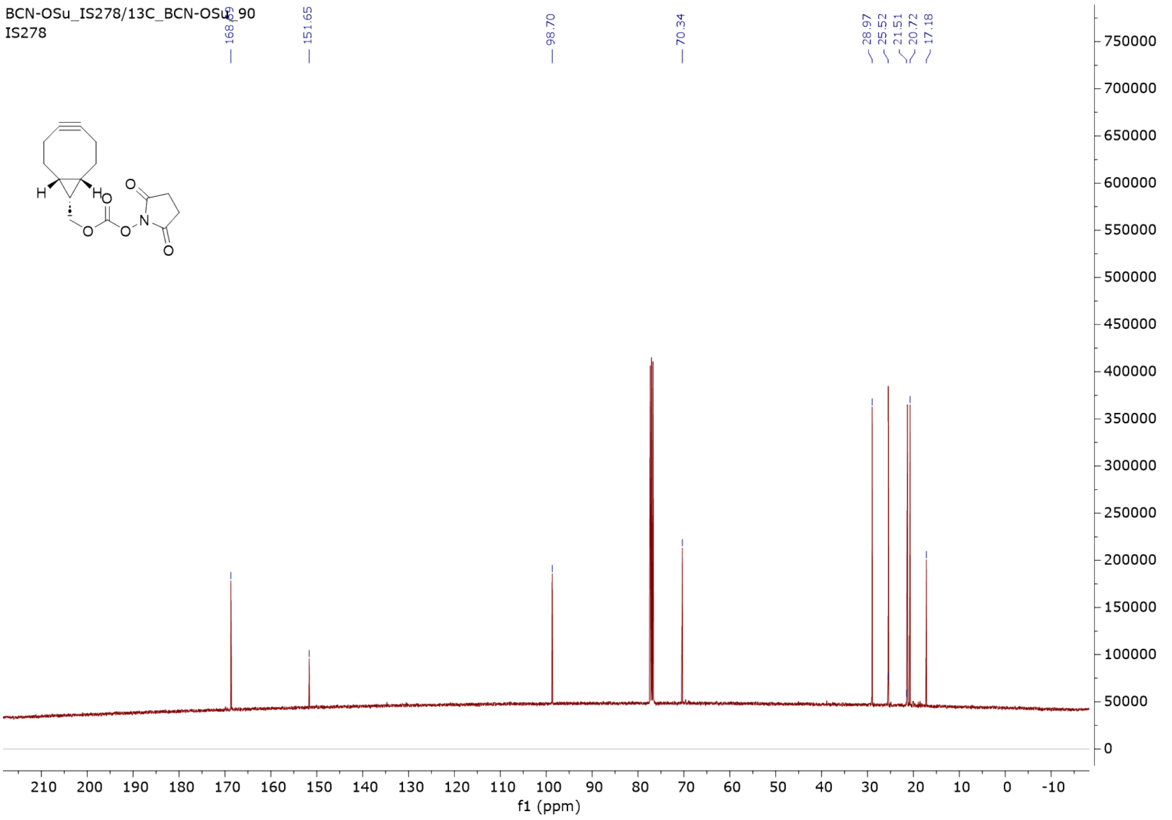

Fig. S18: ^{13}C NMR spectrum of BCN-OSu.

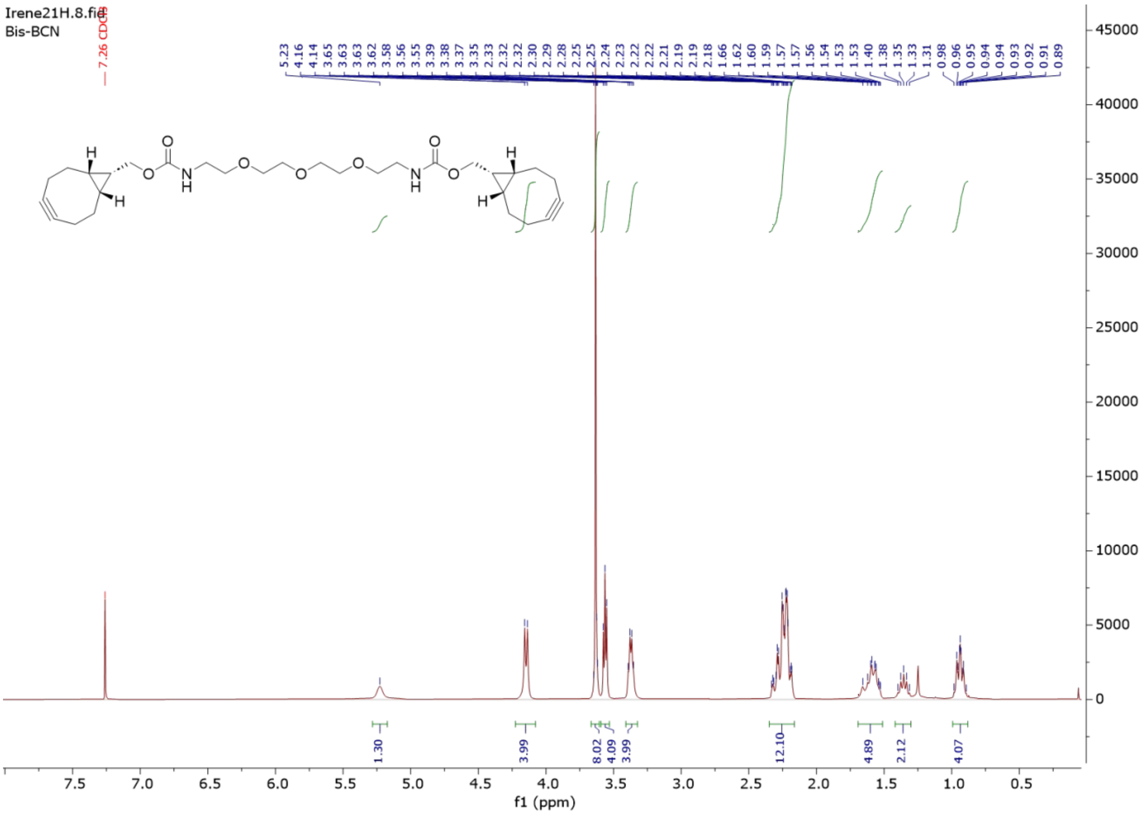

Fig. S19: ^{1}H NMR spectrum of bis-BCN(PEG$_3$) (**1**).

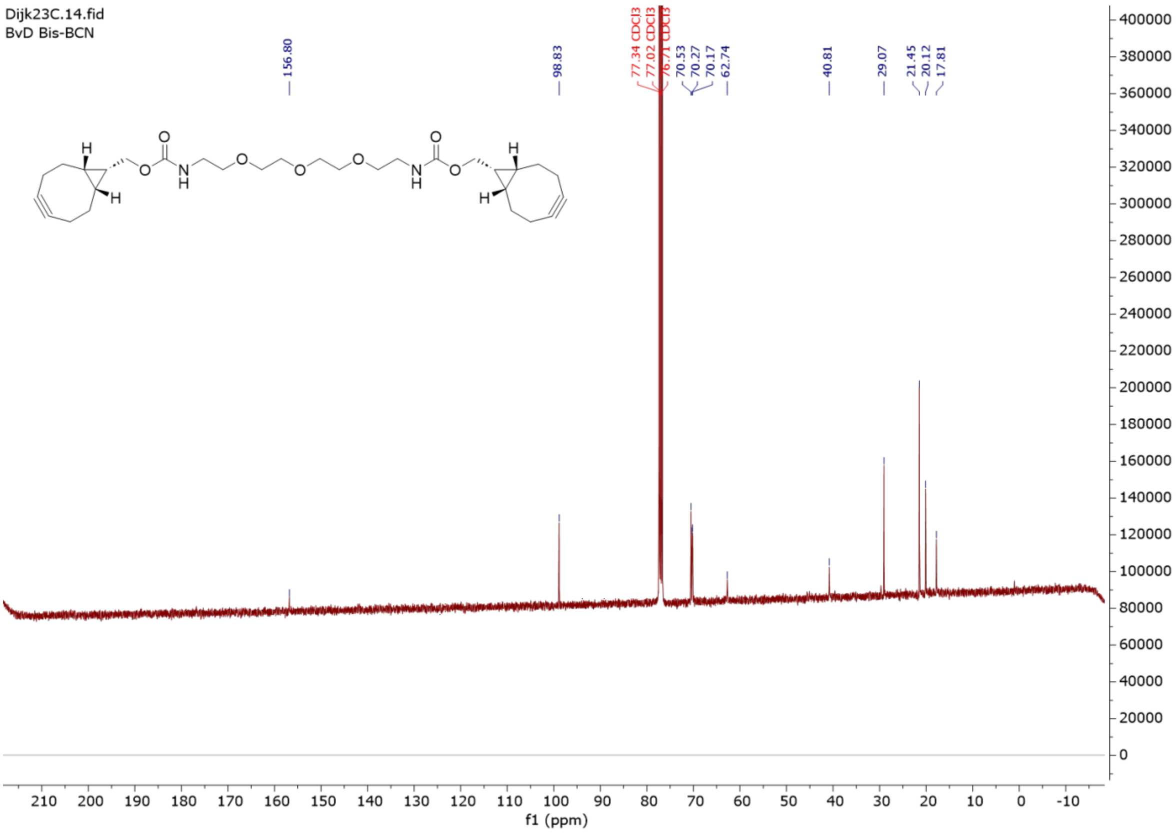

Fig. S20: ^{13}C NMR spectrum of bis-BCN(PEG$_3$) (**1**).

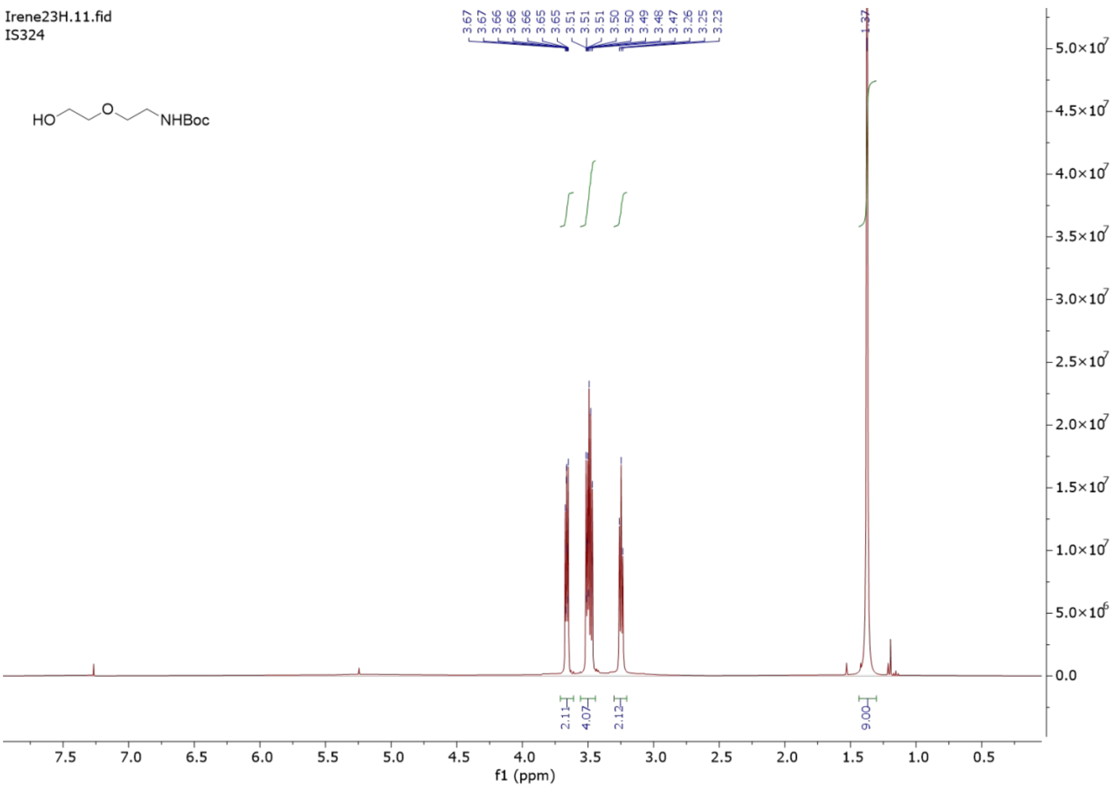

Fig. S21: ^{1}H NMR spectrum of *tert*-butyl (2-(2-hydroxyethoxy)ethyl)carbamate (**a**).

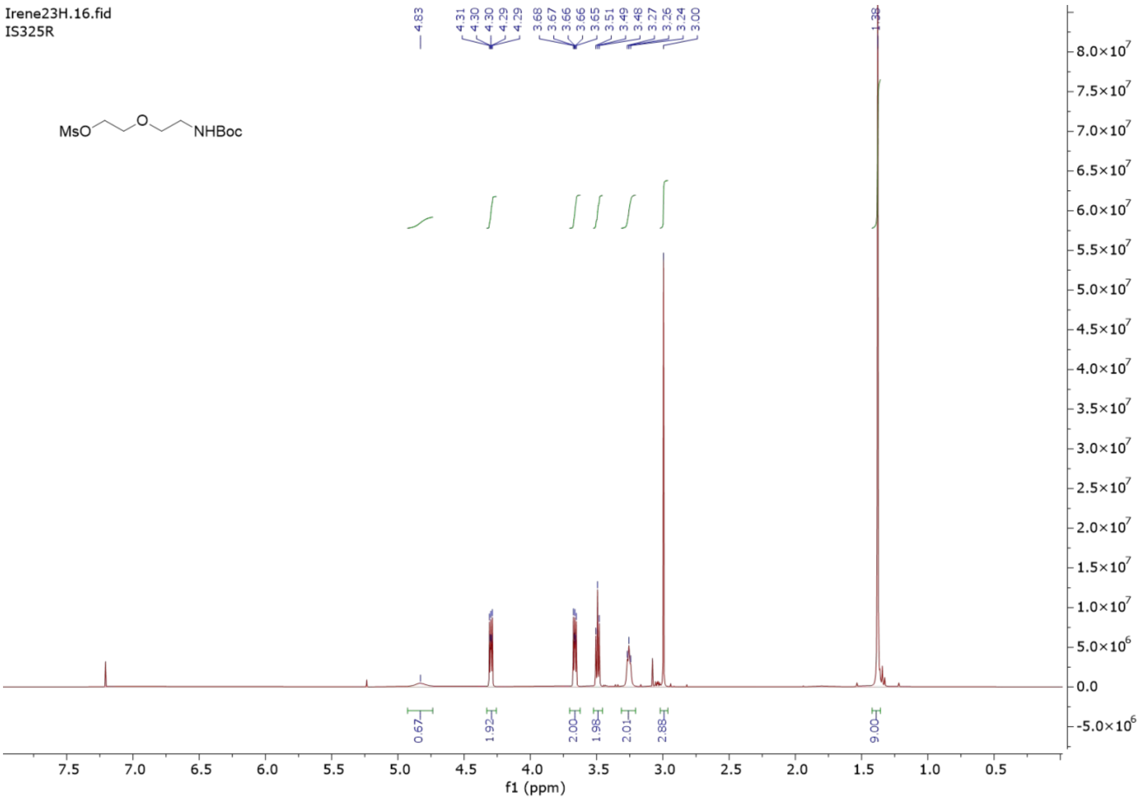

Fig. S22: ^{1}H NMR spectrum of 2-(2-((*tert*-butoxycarbonyl)amino)ethoxy)ethylmethanesulfonate (**b**).

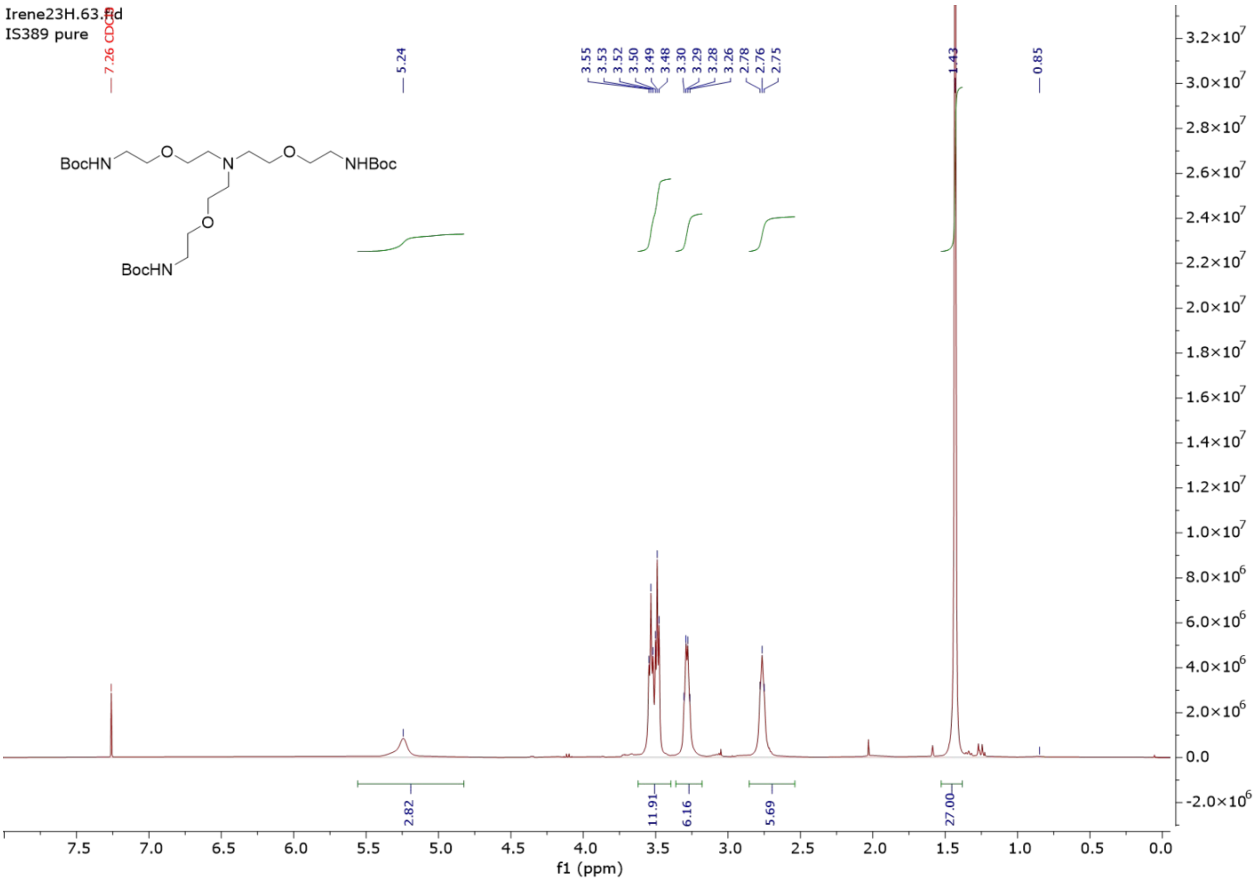

Fig. S23: ^{1}H NMR spectrum of tri-*tert*-butyl-carbamate (**c**).

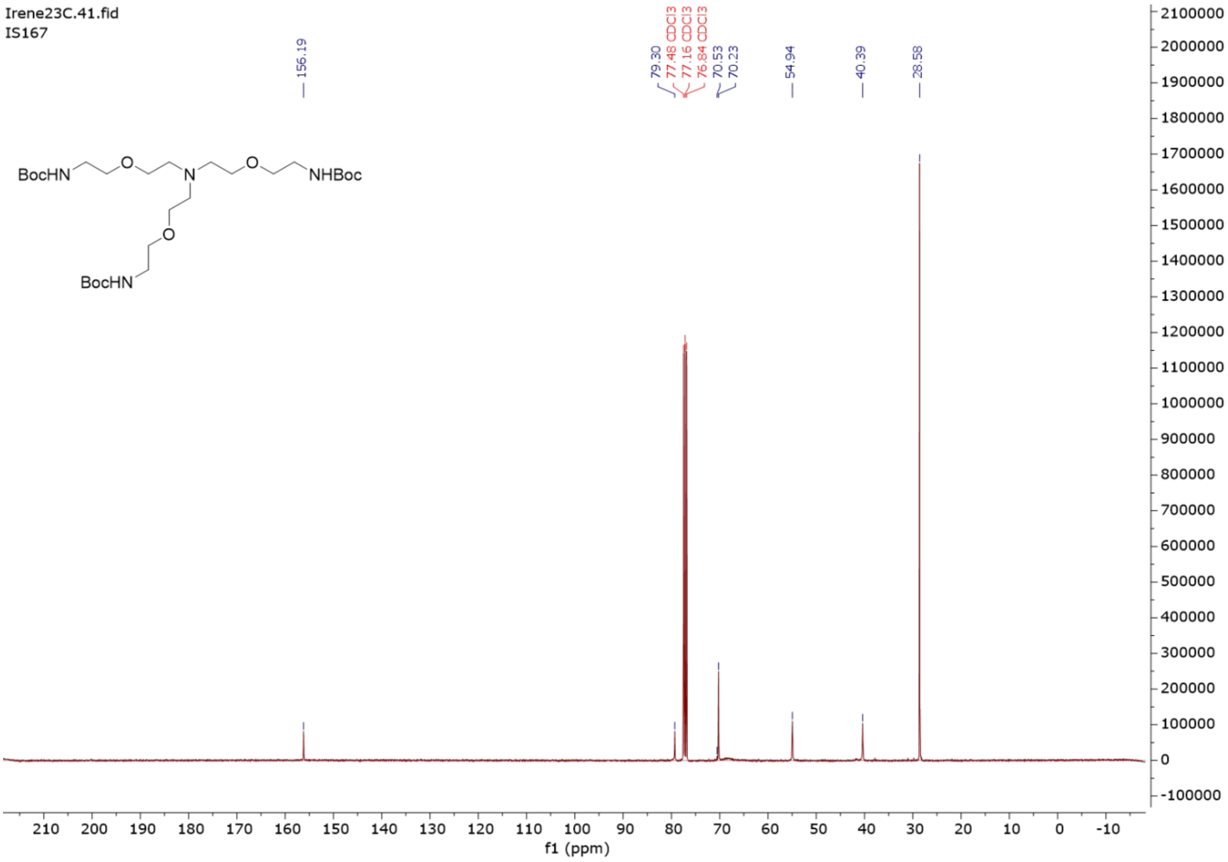

Fig. S24: ^{13}C NMR spectrum of tri-*tert*-butyl-carbamate (**c**).

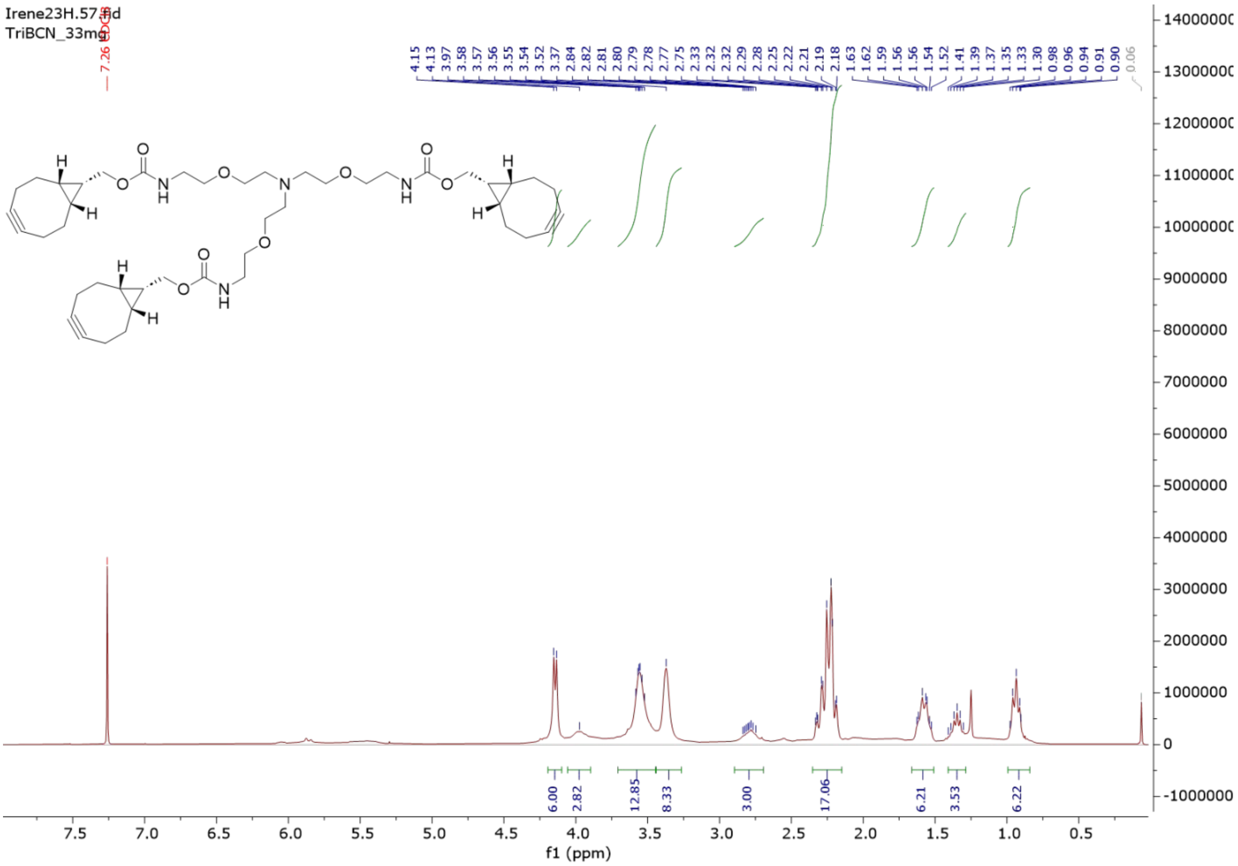

Fig. S25: ^{1}H NMR spectrum of tri-BCN (**2**).

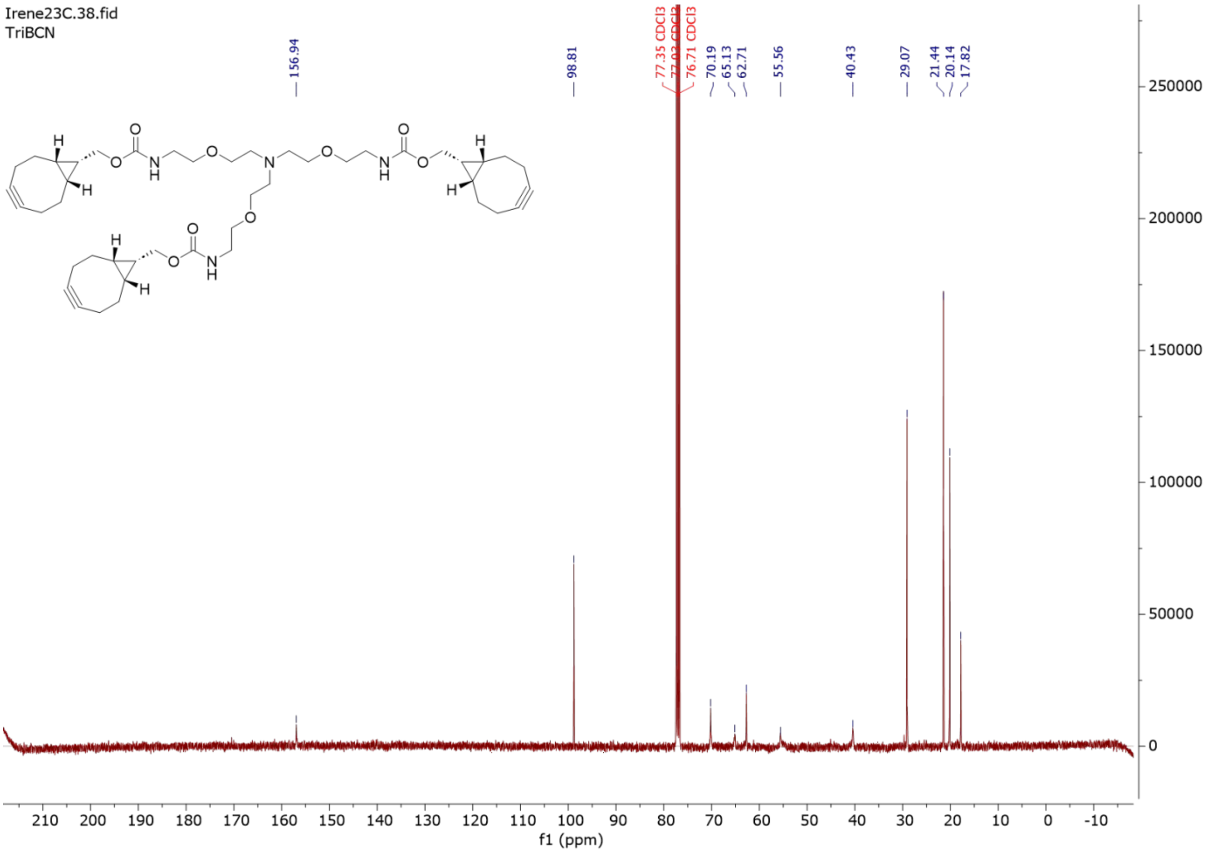

Fig. S26: ^{13}C NMR spectrum of tri-BCN (**2**).

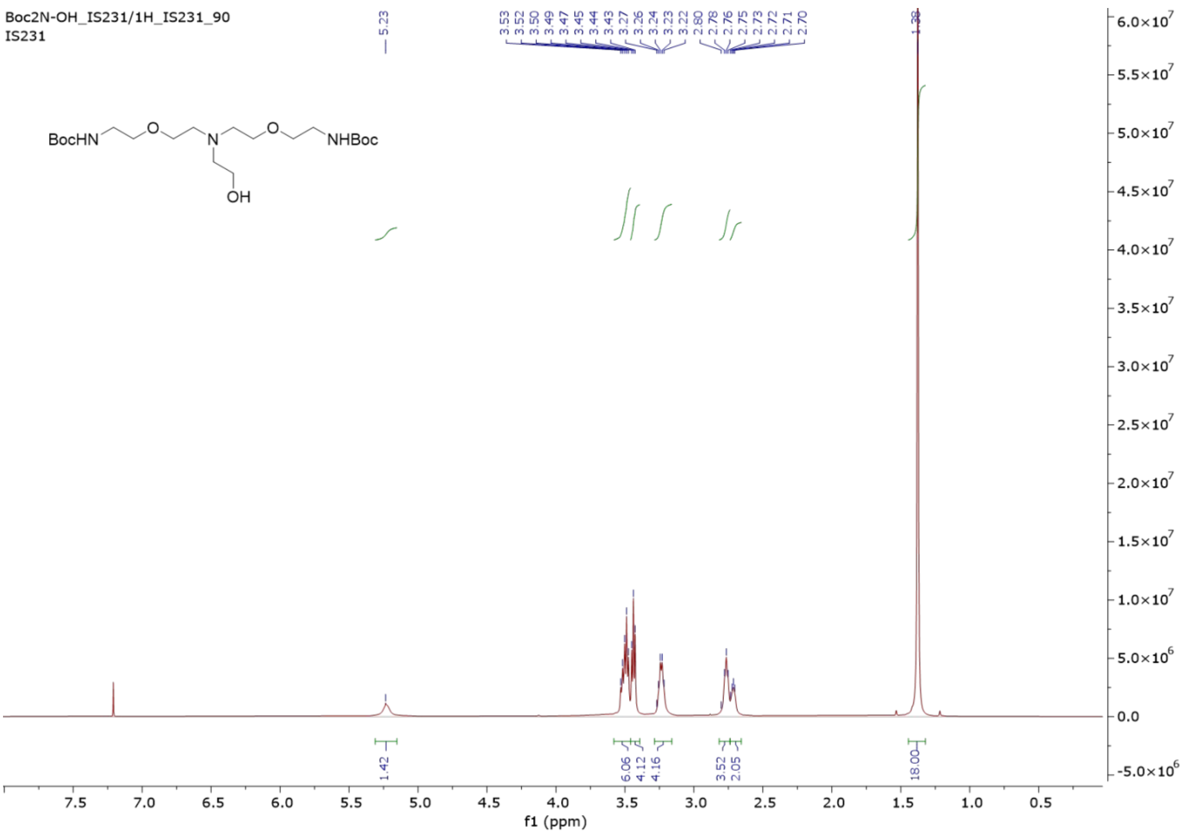

Fig. S27: ^{1}H NMR spectrum of di-*tert*-butyl alcohol (**d**).

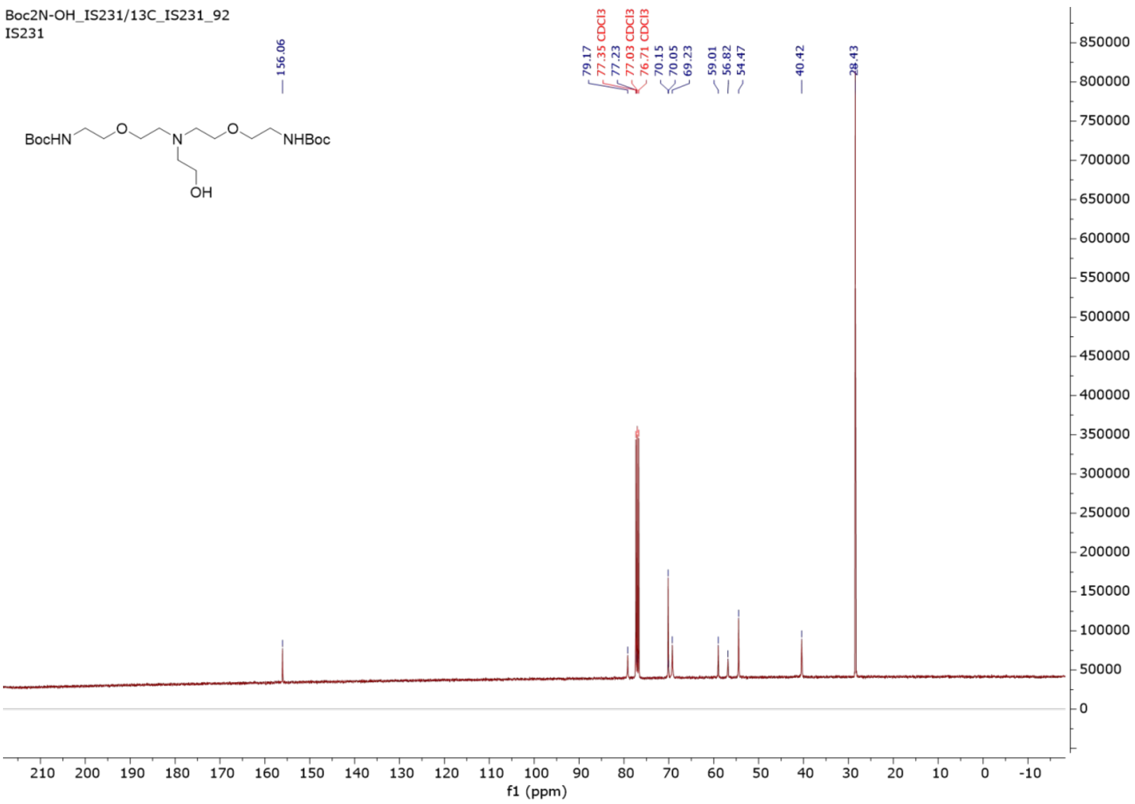

Fig. S28: ^{13}C NMR spectrum of di-*tert*-butyl alcohol (**d**).

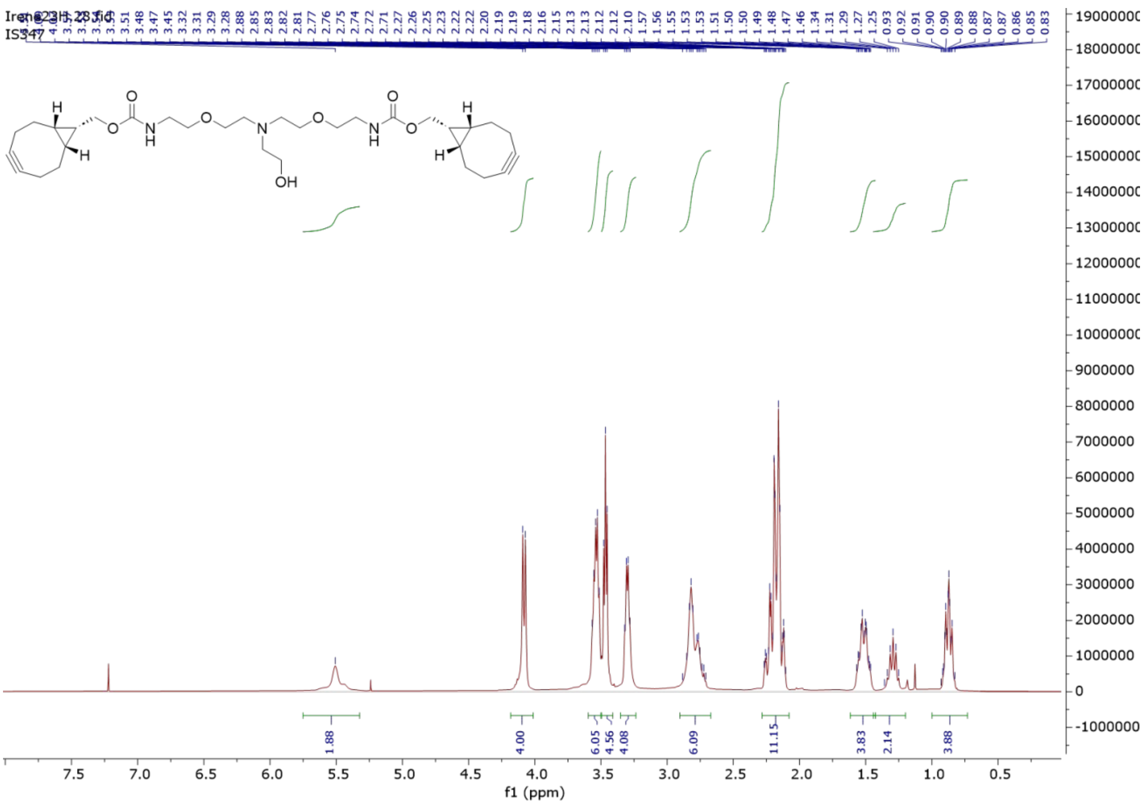

Fig. S29: ^{1}H NMR spectrum of bis-BCN-alcohol (**e**).

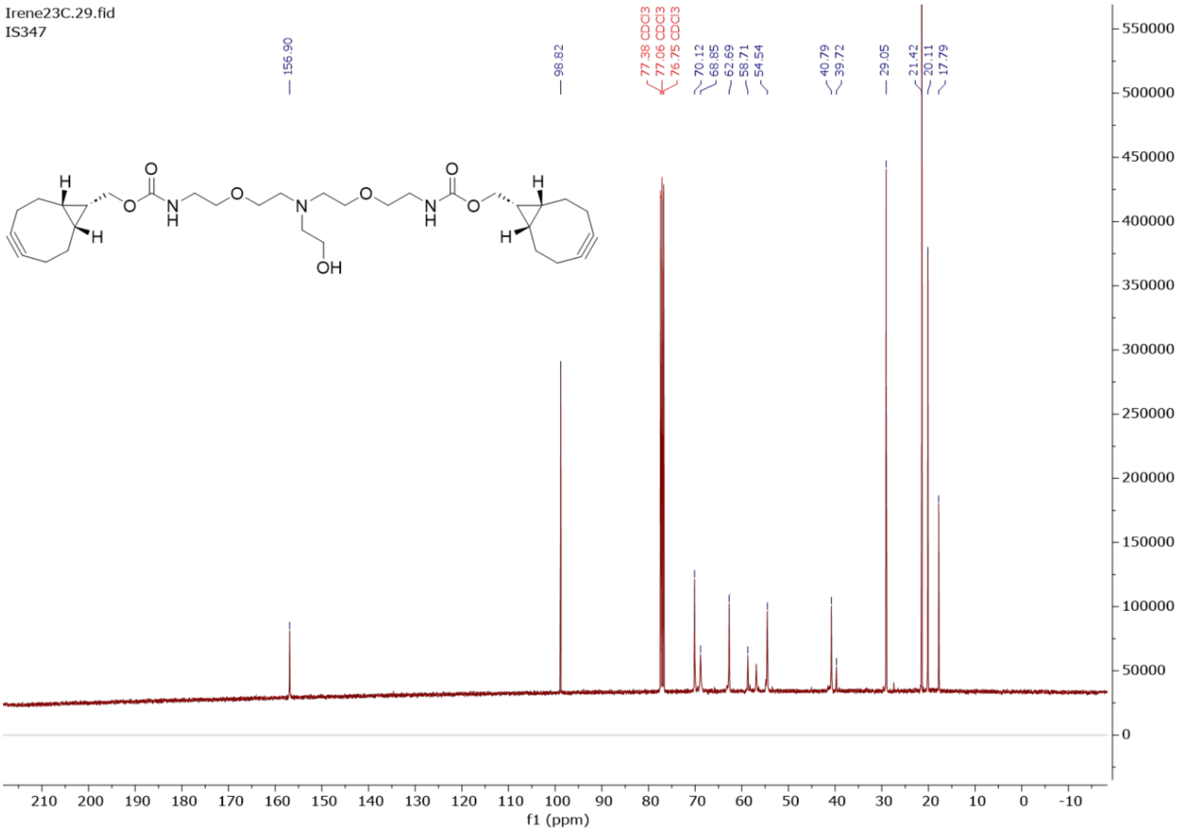

Fig. S30: ^{13}C NMR spectrum of bis-BCN-alcohol (**e**).

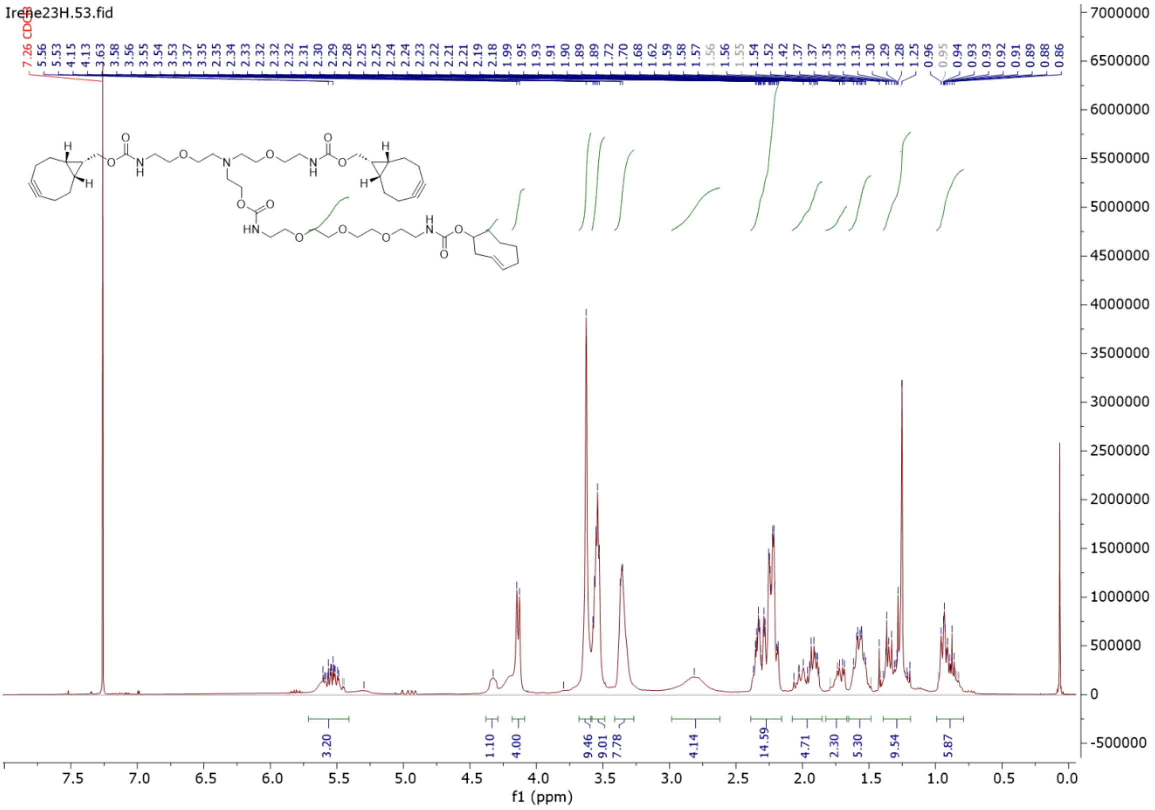

Fig. S31: ^{1}H NMR spectrum of BCN$_2$-TCO (**3**).

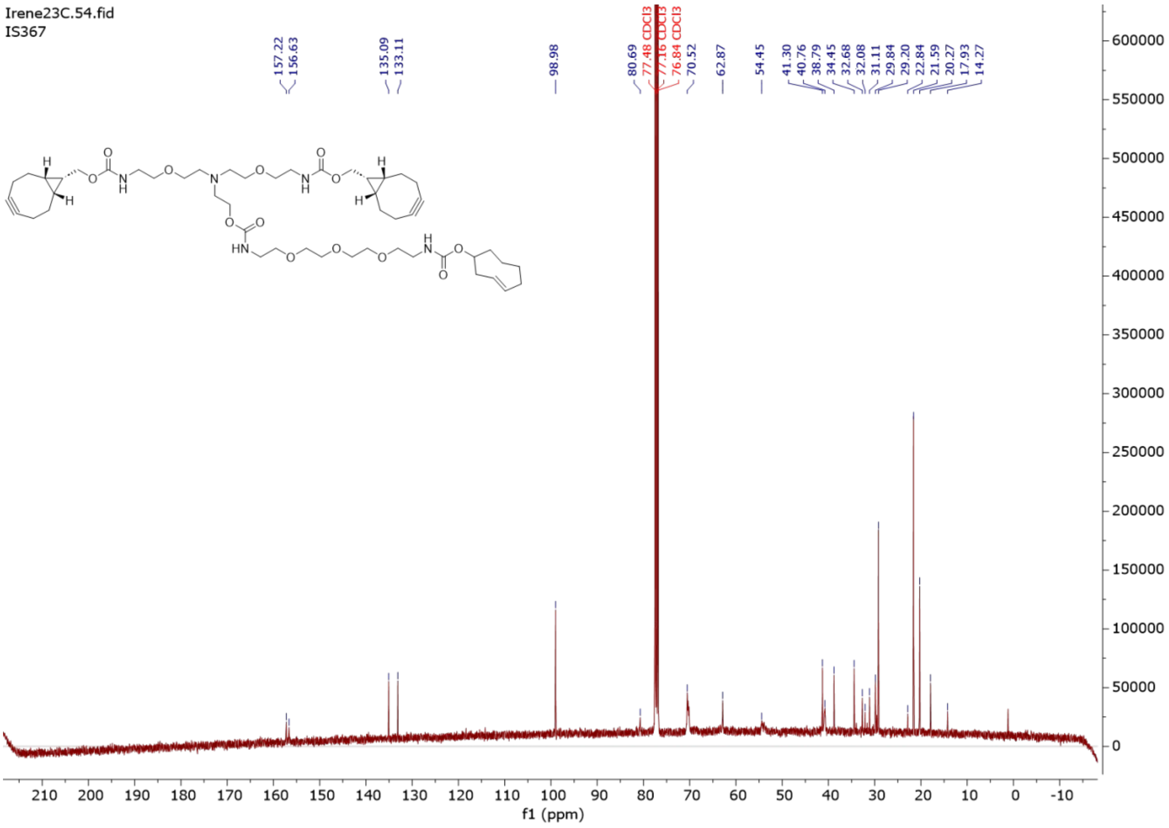

Fig. S32: ^{13}C NMR spectrum of BCN$_2$-TCO (**3**).

LC-MS data

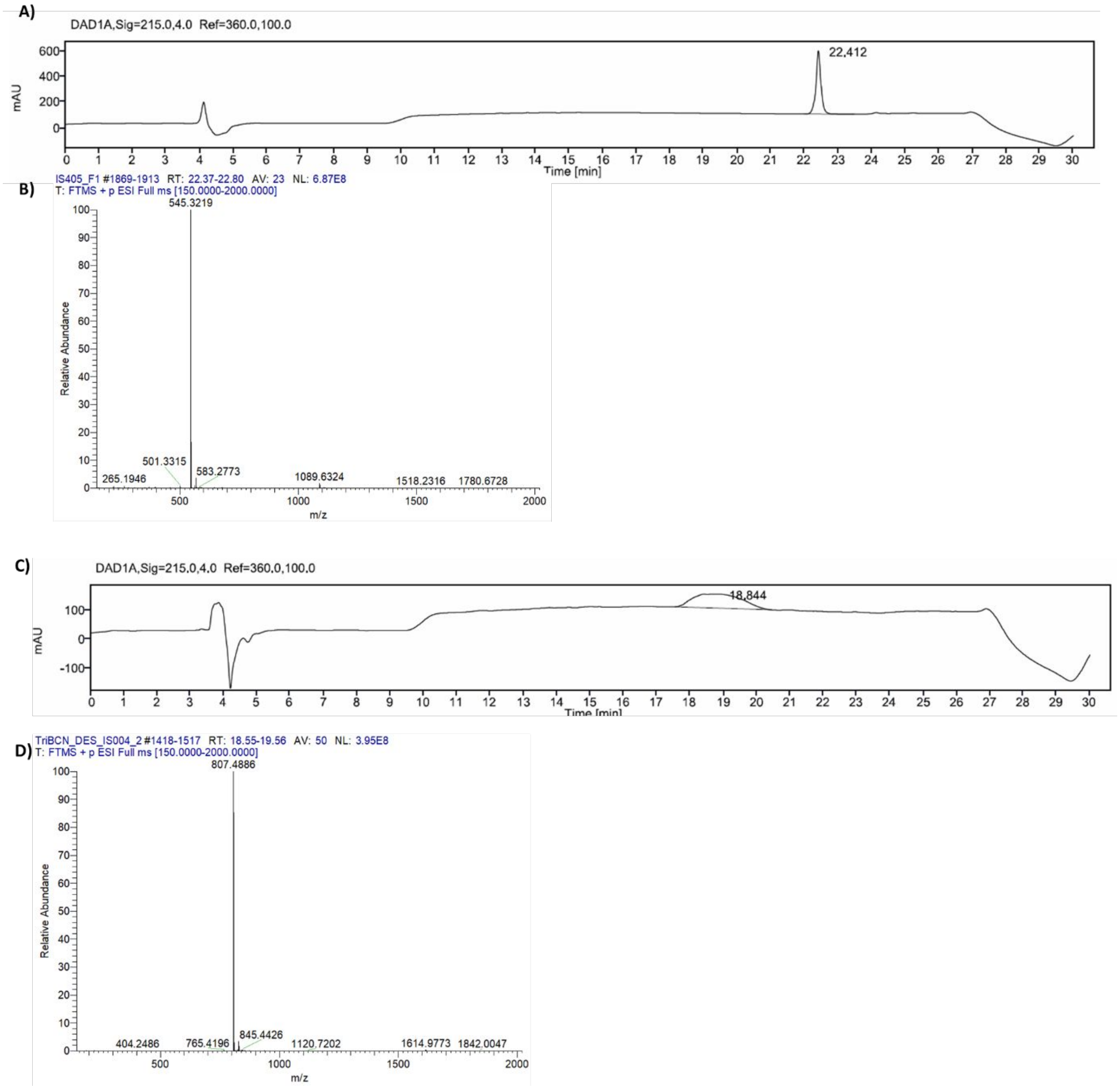

(see next page for caption)

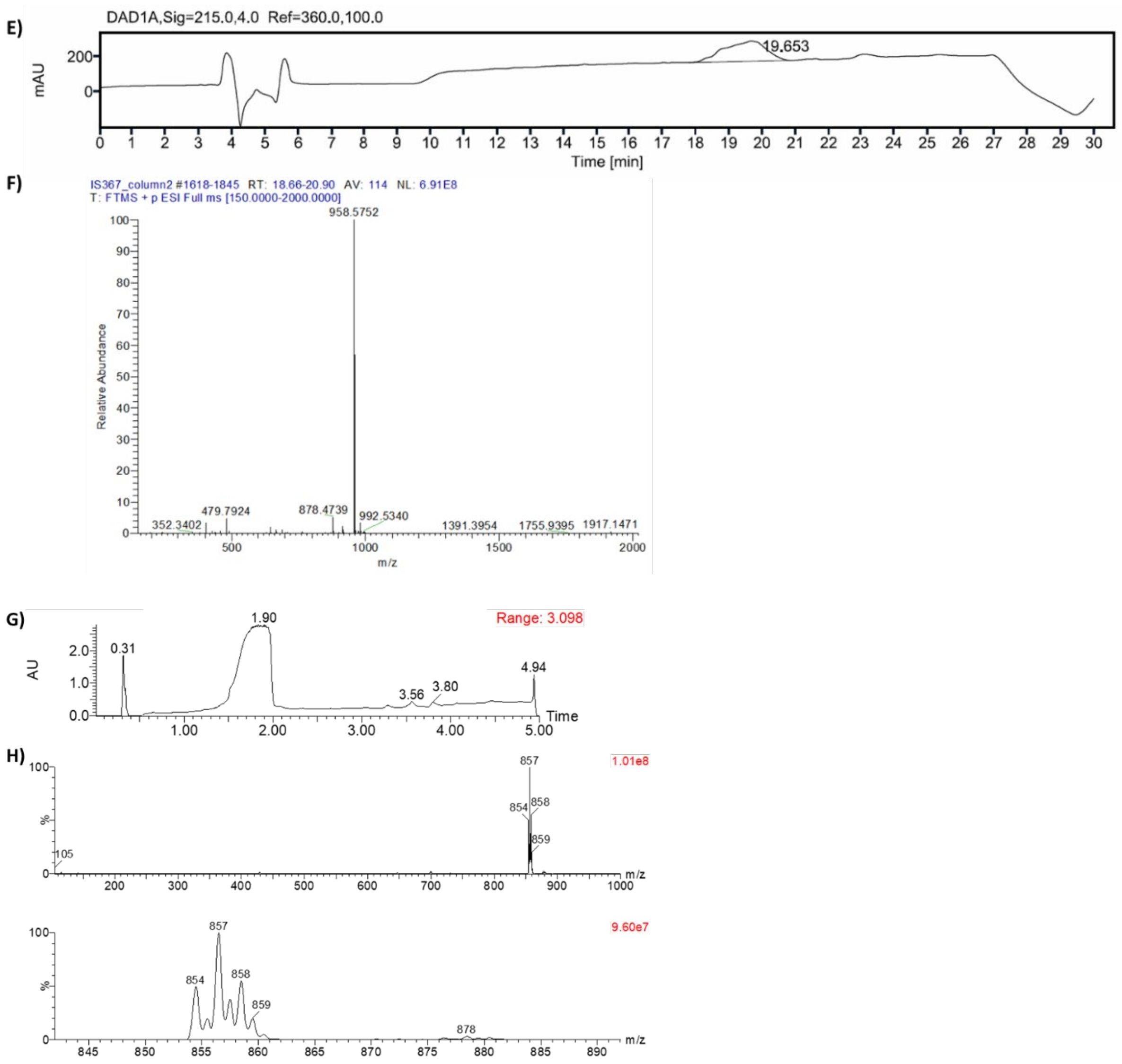

Fig. S33: A) HPLC-UV trace and B) MS trace of bis-BCN (**1**), C) HPLC-UV trace and D) MS trace of tri-BCN (**2**), E) HPLC-UV trace and F) MS trace of BCN_2-TCO (**3**), G) HPLC-UV trace and H) MS trace of Br_2-PD-MeTz (**4**).

Appendix

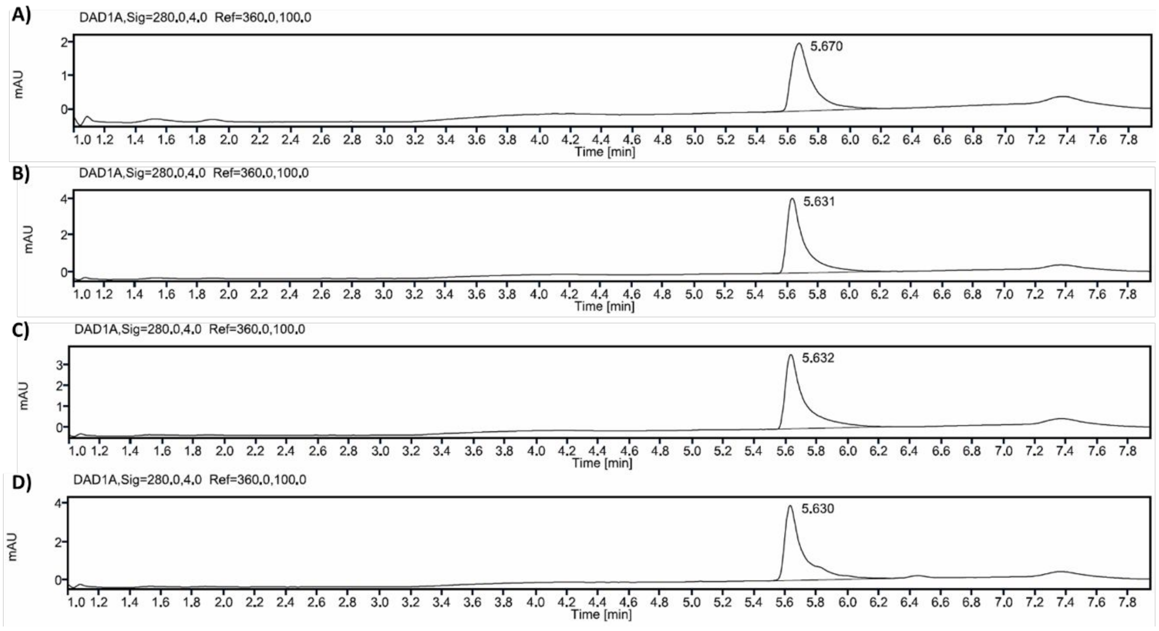

Fig. A1: RP-HPLC trace of intact A) tras[LC]G_4Y, B) mAb construct **7**, mAb construct **8** and mAb construct **9**.

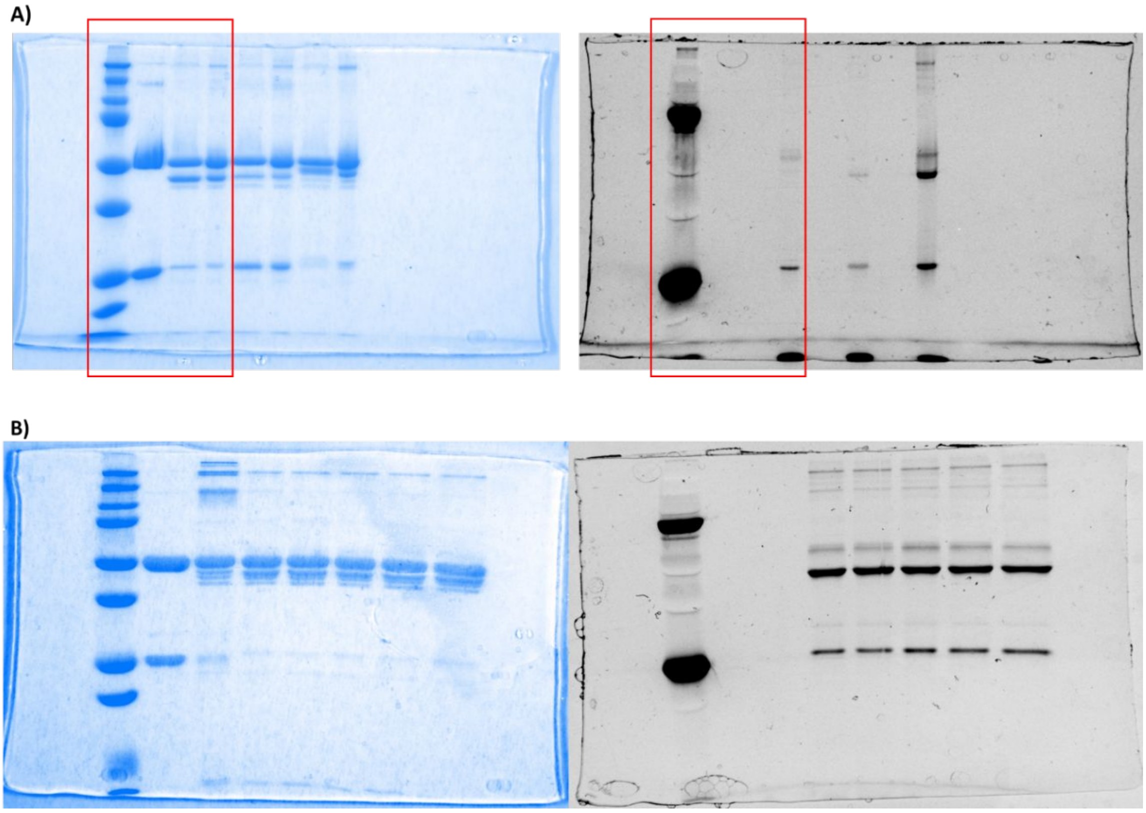

C)

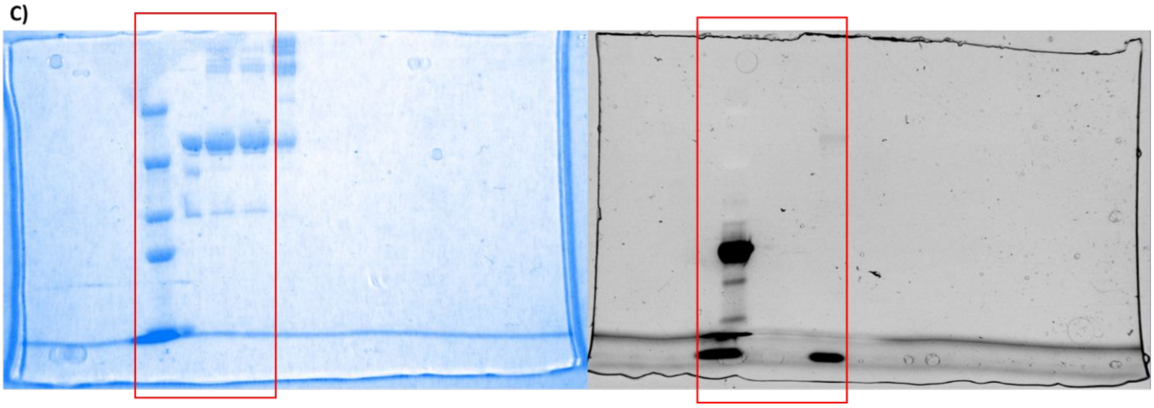

D)

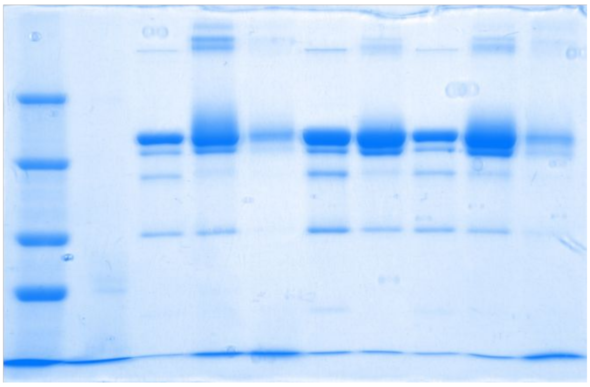

Fig. A2: Full SDS-PAGE gel images of A) S1H, B) S3E, C) S3F and D) S1I and S2F.

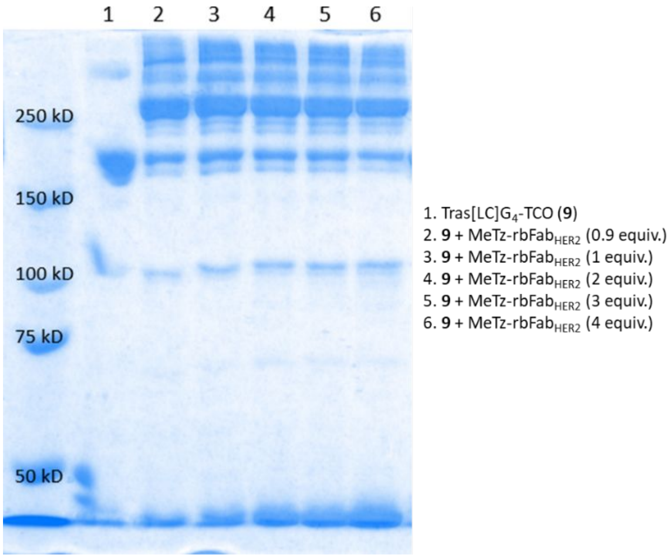

Fig. A3: 6% non-reducing SDS-PAGE of preparation of 2:1 HER2xHER2 construct (equivalence optimization).

References

(51) Marty, M. T.; Baldwin, A. J.; Marklund, E. G.; Hochberg, G. K. A.; Benesch, J. L. P.; Robinson, C. V. Bayesian Deconvolution of Mass and Ion Mobility Spectra: From Binary Interactions to Polydisperse Ensembles. *Anal. Chem.* **2015**, *87*, 4370-4376. DOI: 10.1021/acs.analchem.5b00140.

(52) Yoo, D. Y.; Barros, S. A.; Brown, G. C.; Rabot, C.; Bar-Sagi, D.; Arora, P. S. Macropinocytosis as a Key Determinant of Peptidomimetic Uptake in Cancer Cells. *J. Am. Chem. Soc.* **2020**, *142*, 14461-14471. DOI: 10.1021/jacs.0c02109.
